# Site-specific integration in CHO cells mediated by CRISPR/Cas9 and homology-directed DNA repair pathway

**DOI:** 10.1038/srep08572

**Published:** 2015-02-25

**Authors:** Jae Seong Lee, Thomas Beuchert Kallehauge, Lasse Ebdrup Pedersen, Helene Faustrup Kildegaard

**Affiliations:** 1 The Novo Nordisk Foundation Center for Biosustainability, Technical University of Denmark, 2970 Hørsholm, Denmark

## Abstract

Chinese hamster ovary (CHO) cells are the most widely used mammalian hosts for production of therapeutic proteins. However, development of recombinant CHO cell lines has been hampered by unstable and variable transgene expression caused by random integration. Here we demonstrate efficient targeted gene integration into site-specific loci in CHO cells using CRISPR/Cas9 genome editing system and compatible donor plasmid harboring a gene of interest (GOI) and short homology arms. This strategy has enabled precise insertion of a 3.7-kb gene expression cassette at defined loci in CHO cells following a simple drug-selection, resulting in homogeneous transgene expression. Taken together, the results displayed here can help pave the way for the targeting of GOI to specific loci in CHO cells, increasing the likelihood of generating isogenic cell lines with consistent protein production.

Chinese hamster ovary (CHO) cells have been used as the predominant workhorses for production of recombinant therapeutic proteins with complex glycoforms^1^,^2^. Traditionally, development of recombinant CHO (rCHO) cell lines relies on random integration of a gene of interest (GOI) into the genome, followed by selection of cells carrying the transgene^1^,^2^. However, lack of control of gene insertion can give rise to unwanted phenotypic heterogeneity due to the varying accessibility of integration sites for gene expression – termed the position effect variation^2^,^3^. In addition, gene amplification methods are traditionally used to increase expression. As a result, these cell lines are often unstable and show reduced production over time^4^. Due to this variation in expression and genomic composition, subsequent screening of multiple clones is necessary to select proper clones suitable for high and stable expression of recombinant proteins^1^,^2^. In this article, we show that targeting of transgenes into specific desirable sites in the CHO genome in a controlled manner reduces the variation in expression, generating a uniform population with stable transgene expression.

In order to circumvent uncontrollable gene insertion during rCHO cell line construction, different approaches have been applied for site-directed integration of transgenes using site-specific recombinases, which includes Cre/loxP system, Flp/FRT system, and phiC31/R4 integrases^5^,^6^,^7^. However, these systems are limited by the need of a prior establishment of platform cell lines with the insertion of recombination site into random or a limited number of specific genomic regions, which is a prerequisite for retargeting to generate cell lines expressing the GOI.

With draft genomes of several CHO cell lines recently being made available^8^,^9^, it is now possible to efficiently engineer the genomic sequence of CHO cells with engineered nucleases. Customized nucleases such as zinc-finger nucleases (ZFNs), transcription activator-like effector nucleases (TALENs), and clustered regularly interspaced short palindromic repeats (CRISPR)-associated (Cas) RNA guided nucleases are technologies readily available to induce targeted insertion/deletion (indel) mutations or precise sequence changes in a broad range of organisms and cell types (reviewed in Ref. 10). Upon site-specific DNA double-strand breaks (DSBs) induced by engineered nucleases, the target locus will typically be repaired by one of two major DNA damage repair pathway: nonhomologous end-joining (NHEJ) or homology-directed repair (HDR). Compared with the error-prone NHEJ, which can result in efficient indel mutation generation subsequently leading to knockout of target locus, HDR can be used to modify endogenous loci precisely in the presence of homologous pieces of endogenous/exogenous DNA. Although HDR is typically observed at a lower or more variable frequency than NHEJ, HDR can be leveraged to generate targeted integrations with engineered nucleases in mammalian cells^11^,^12^,^13^,^14^,^15^. Notably, CHO cells have previously been described as a cell type recalcitrant to homology-based integration of large DNA constructs, thus targeted integration in CHO cells has been confined to NHEJ based approaches^16^,^17^.

Compared with protein-based genome editing tools with customizable DNA binding specificities, such as ZFNs and TALENS, the newer CRISPR/Cas9 platform is based on simple base-pairing between an engineered RNA and the targeted genomic site, which enables rapid design, ease of use, and low costs^18^. The CRISPR/Cas9 system, which was initially identified in the bacterial immune system, is composed of two RNA elements, CRISPR-RNA (crRNA) and trans-activating crRNA (tracrRNA), together with the Cas9 nuclease^14^,^19^. The active cleavage-complex is generated by base-pairing between the genomic sequence and crRNA, followed by hybridization of the tracrRNA to the crRNA. Subsequently, the Cas9 nuclease associates with the DNA:RNA complex to form the active cleavage complex, which in turn will generate site specific DSBs at the genomic locus^19^. In recent studies, the original type II CRISPR system from *S. pyogenes* was modified to induce targeted genome editing in eukaryotic cells by fusing the two RNA complexes termed single guide RNA (sgRNA)^11^,^14^,^19^. We previously developed a CRISPR/Cas9 system optimized for CHO cells, and demonstrated high efficiency of indels generated in CHO cells up to 47.3%^20^. Assuming that the high rate of DSB introduction will induce DNA damage repair pathways, and the concurrent introduction of a donor plasmid will be used as the repair template, the targeted integration at specific genomic sites can be feasible with high fidelity.

Here we demonstrate efficient targeted gene integration into site-specific loci in CHO cells using CRISPR/Cas9 genome editing system and compatible donor plasmid harboring short-homology arms, GOI, and a fluorescent marker gene as an indicator of random integration. Simultaneous introduction of active sgRNAs, Cas9 nucleases, and donor plasmid enabled insertion of a 3.7-kb gene expression cassette at three different loci in CHO cells, following a simple drug-selection. A low level of off-target mutation in most potential off-target sites and homogeneous expression level of GOI was observed in targeted integrants, which supports the robustness and efficiency of the current strategy.

## Results

### Development of a HDR mediated targeted integration platform for CHO

**Figure 1:**
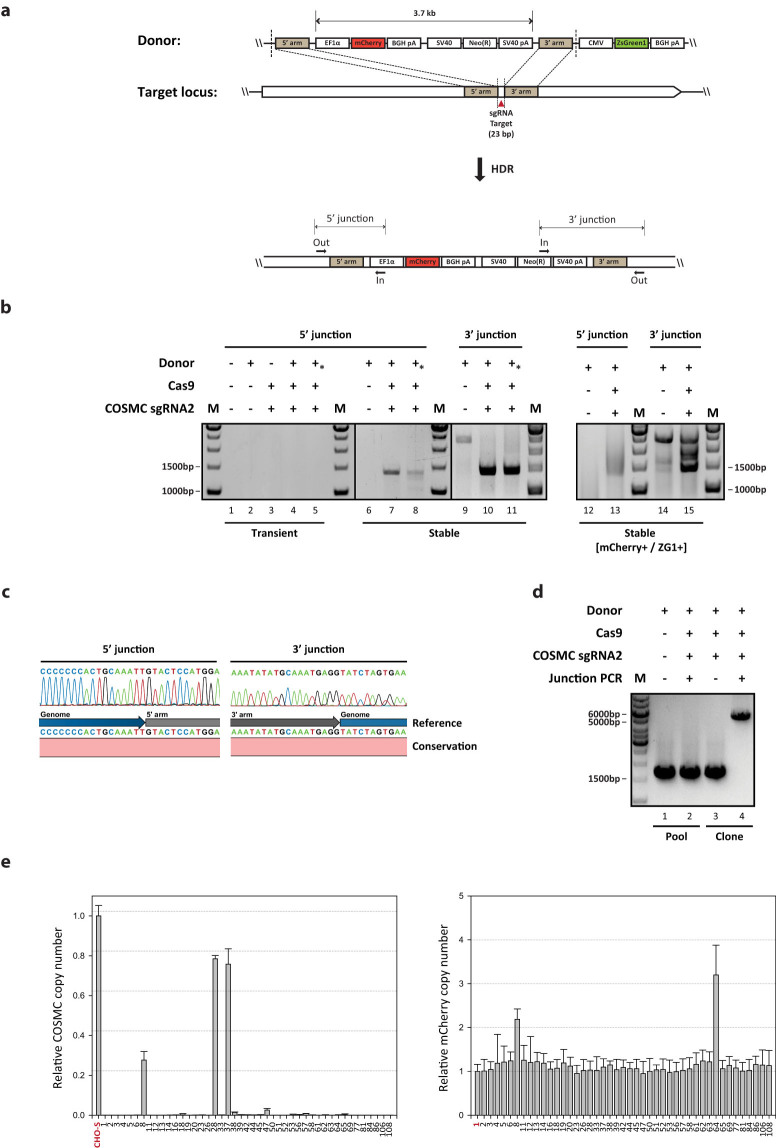
Targeted integration into *COSMC* locus using CRISPR/Cas9. (a) Schematic illustration of the targeting strategy for the specific locus of interest. Donor plasmid consists of three parts: short homology arms flanking sgRNA target site cleaved by Cas9 (red triangle), mCherry and neomycin resistance gene expression cassettes inside homology arms, and ZsGreen1-DR expression cassette outside homology arms. Upon DSBs induced by CRISPR/Cas9, HDR-mediated repair can be used to insert a total size of 3.7 kb of expression cassettes through recombination of the target locus with donor plasmids. Primer position for 5′/3′ junction PCR is indicated. (b) Agarose gel of 5′/3′ junction PCR on transiently transfected cells and stable cell pools. An asterisk indicates the use of linearized donor plasmid. M, 1 kb DNA ladder (c) Sanger sequencing of the 5′/3′ junction PCR amplicons. Amplicons from the stable cell pool were purified and directly sequenced after PCR amplification. The chromatogram sequence of junction PCR amplicon was compared with the reference sequence at the genome-donor boundaries. (d) Agarose gel of out-out PCR results of stable cell pools or clonal cells. Primer pairs annealing to genomic DNA region were used resulting in PCR products of either wild type (1.6 kb) or targeted integration (5.3 kb). (e) Relative copy number of *COSMC* and *mCherry* regions in clonal cells. Each plot shows the relative copy number of each region in comparison to the reference sample. Genomic DNA of wild type CHO-S and Clone #1 and was used as the reference for *COSMC* and *mCherry* region, respectively (shown in red). The error bars represent the standard deviations (n ≥ 3).

To facilitate improved construction of rCHO cell lines, we aimed at developing a CRISPR and HDR mediated targeted integration system to obtain controlled and precise integration of transgenes. The system is based on donor plasmids harboring short homology arms, which flank the Cas9 cleavable sgRNA target site, used as the integration sites (Fig. 1a). The donor plasmids were constructed as follows: Regardless of loci and sgRNAs, the homology arms were designed to be exactly next to the whole 23 bp of sgRNA genomic target sequences (Supplementary Table 1). The length of each 5′ and 3′ homology arm was set at 750 bp unless otherwise mentioned. An mCherry expression cassette and a neomycin resistance gene expression cassette were placed within the homology arms. A ZsGreen1-DR expression cassette was placed outside the homology arms to detect random integration events. Upon correct targeting of the donor plasmids to the desired locus via HDR, cells will lose ZsGreen1-DR expression and only express mCherry together with neomycin resistance. If cells express both mCherry and ZsGreen1-DR after neomycin selection, random integration of the donor plasmid has occurred. This assumption was applied in order to enrich for targeted integrants by excluding clones expressing ZsGreen1-DR. To evaluate correct integration by HDR, 5′/3′ junction PCR amplicons were designed for each target site (Fig. 1a). We targeted a total of four sites in three different genomic loci of CHO cells; one site in *C1GALT1C1* (*COSMC*) encoding the C1GALT1-specific chaperone 1, two sites in *Mgat1* encoding the mannosyl (alpha-1,3-)-glycoprotein beta-1,2-N-acetylglucosaminyltransferase, and one site in *LdhA* encoding the lactate dehydrogenase A.

### Precise targeted integration of transgene at the *COSMC* locus in CHO cells

The *COSMC* locus has previously been effectively modified by CRISPR/Cas9 system in CHO cells^20^. Thus, this locus was chosen for initial validation of the targeted integration strategy. The region targeted by sgRNA2, which gave rise to high indel efficiency in CHO-K1 cells in our previous study^20^, was selected as the integration site. Based on this site, 5′ and 3′ homology arms were designed to generate the targeting donor plasmid (Fig. 1a). CHO-S cells were transfected with the vectors encoding CHO codon optimized Cas9, COSMC sgRNA2, and donor plasmid. Genomic DNA was extracted either three days after transfection (transient expression) or two weeks after G418 selection (stable expression). No integration events were detected in the transiently transfected pools by the 5′/3′ junction PCR (Fig. 1b, Lane 1–5). In the stable selected pools, both 5′ and 3′ junction PCR verified targeted integration of donor DNA into the *COSMC* locus (Fig. 1b, Lane 7 and 10). In agreement with a previous observation^16^, linearization of the donor DNA outside of the homology arms resulted in fainter PCR positive bands from 5′/3′ junction PCR compared with those from circular donor DNA (Fig. 1b, Lane 8 and 11). Stepwise decreasing the length of 5′ and 3′ homology arms flanking the same *COSMC* integration site from 750 bp to 100 bp indicated that homology arms with a length down to 250 bp were capable of mediating homology-based targeted integration, although the PCR band intensity was gradually decreased (Supplementary Fig. 1). In accordance with the assumption that cells expressing both ZsGreen1-DR and mCherry were the result of random integration, stable cells expressing both mCherry and ZsGreen1-DR, which were isolated by FACS, showed incomplete and faint junction PCR positive bands indicating a mixture of integration events. (Fig. 1b, Lane 13 and 15). To verify precise integration into the genome, the 5′/3′ genome-donor boundaries were sequenced (Fig. 1c). Indeed, the sequences of the 5′/3′ junction PCR amplicons confirmed precise insertion of the target expression cassette into the *COSMC* locus. We then screened 588 G418-resistant clones and isolated 138 clones, which displayed homogeneous expression of mCherry. 23 clones were excluded due to very slow growth resulting in 115 clones, which were further characterized. 83 out of 115 (72.2%) were 5′/3′ junction PCR positive (Table 1). Out-out PCR with primers specific for genomic sites revealed that 70 out of 83 junction PCR positive clones (84.3%, i.e. 60.9% in total) were correctly targeted with the intact target integration unit, generating expected size of amplicon (wild type amplicon: 1.6 kb + target integration unit: 3.7 kb ≈ 5.3 kb; Fig. 1d, Lane 4, Table 1). As a result, a targeting efficiency of 27.8% was obtained based on the percentage of out-out PCR positive clones in mCherry only expressing clones and a proportion of the mCherry only expressing cells in the stable cell pool (Table 1). These 70 out-out PCR positive clones were expanded and adapted to grow in suspension. A total of 45 clones were recovered, followed by further characterization. 42 out of 45 clones had complete disruptions of the *COSMC* locus, as indicated by qPCR-based relative copy number analysis compared with genomic DNA from CHO-S wild type cells (Fig. 1e, left panel). Assuming that gene copies must be integer values, the value of relative copy number is expected to be 0, 0.5, or 1 for targeted clonal cells. Three clones without these values were therefore not considered as clonal cells. Relative mCherry copy number result amongst selected clones revealed that most clones harbored the same number of mCherry transgene, except two clones with 2- or 3-fold higher number (Fig. 1e, right panel). Successful targeting of transgenes at the *COSMC* locus validated our integration method, and prompted us to apply to different genomic loci showing distinct properties.

### Application of targeted integration platform to the functional hemizygous locus*, Mgat1*

**Figure 2:**
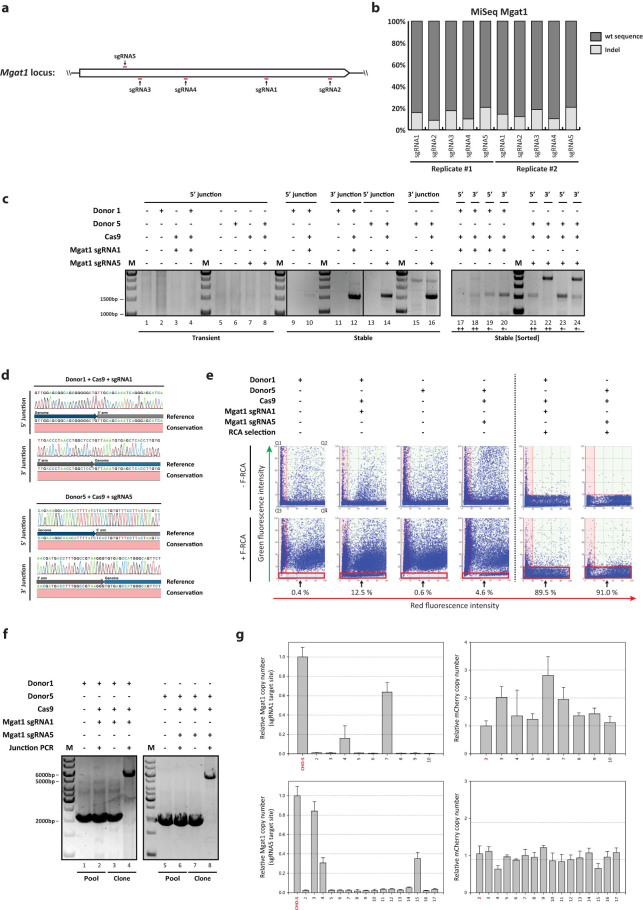
Targeted integration into *Mgat1* locus using CRISPR/Cas9. (a) Illustration of the five sgRNA target genomic sites in *Mgat1* locus. (b) Indel frequency in *Mgat1* locus analyzed by deep sequencing. Genomic DNA was extracted 3 days after transfection with plasmids expressing Cas9 gene and sgRNAs. The genomic regions covering sgRNA target sites were amplified, then subjected to Miseq analysis. The percentage of wt and indel sequences are described in the bar plot. The values from control samples transfected only with plasmid expressing Cas9 were subtracted from test samples. (c) Agarose gel of 5′/3′ junction PCR on transiently transfected cells and stable cell pools. (++) Stable cells expressing both mCherry and Zsgreen1-DR; (+-) stable cells expressing only mCherry. (d) Sanger sequencing of the 5′/3′ junction PCR amplicons. Amplicons from the stable cell pools were purified and directly sequenced after PCR amplification. M, 1 kb DNA ladder. (e) Population analysis of *Mgat1* disrupted cells by F-RCA-I staining. Based on red/green fluorescence intensity of stable cell pools, which was further selected with RCA-I or not, alteration of fluorescence intensity was analyzed upon F-RCA-I staining. Each scatter plot was divided by four quadrants, denoted by Q1 to Q4. Q3 and Q4 populations, marked by red squares, represent negative stained cells with F-RCA-I, indicating functional knockout of *Mgat1* locus. Numerals below the red squares show the percentage of Q3 and Q4. (f) Agarose gel of out-out PCR results of stable pools or clonal cells. Primer pairs annealing to genomic DNA region were used resulting in PCR products of either wild type (2.0 kb for sgRNA1 target site; 1.9 kb for sgRNA5 target site) or targeted integration (5.6 kb for sgRNA1 target site; 5.5 kb for sgRNA5 target site). (g) Relative copy number of *Mgat1* and *mCherry* regions in clonal cells, as described in Fig. 1(e). (*Top*) sgRNA1 target site (*Bottom*) sgRNA5 target site.

The *Mgat1* locus was selected as a second integration site to test the applicability of the targeted integration system at a different genomic locus. Some CHO cell lines contain one functional *Mgat1* allele that renders this locus more prone to be disrupted by chemical mutagenesis or genome editing tools, leading to functional knockout of *Mgat1*^21^,^22^. Since Mgat1 adds N-acetylglucosamine to the Man5GlcNAc2 (Man5) N-glycan structure, *Mgat1*-disrupted CHO cell lines are capable of producing recombinant proteins with terminal mannose residues and show resistance to *Ricinus communis* agglutinin-I (RCA-I), a cytotoxic lectin that binds Man5GlcNAc2^22^. In order to select efficient sgRNA target sites for the *Mgat1* locus, the indel efficiency of five sgRNAs was analyzed by deep sequencing (Fig. 2a). At post-transfection with Cas9 and sgRNA expression vectors, the five tested sgRNAs generated detectable range of indels from 10.0% to 20.7% in the *Mgat1* locus (Fig. 2b). Two sgRNAs, sgRNA1 and sgRNA5, were selected for targeted integration into the *Mgat1* locus. CHO-S cells were transfected with Cas9, sgRNA, and donor plasmid to generate two cell pools, one for each sgRNA. Junction PCR analysis of the stable selected pools were performed (Fig. 2c, Lane 9–16) and compared with junction PCR analysis on transiently transfected cells (Fig. 2c, Lane 1–8). Similar to the *COSMC* locus, 5′/3′ junction PCR was only positive for the stable pools (Fig. 2c, Lane 10 and 12 for sgRNA1 site; Lane 14 and 16 for sgRNA5 site). Furthermore, stable cells expressing both mCherry and Zsgreen1-DR, denoted by ++, showed less clean and intense bands than cells expressing only mCherry, denoted by +-, which indicated incomplete integration (Fig. 2c, Lane 17–24). The sequencing results of 5′/3′ junction PCR amplicons confirmed precise gene insertion occurring at the *Mgat1* locus (Fig. 2d).

The amount of *Mgat1*-disrupted cells in two stable pools targeted for either sgRNA1 or sgRNA5 site was estimated by analyzing fluorescein labeled RCA-I (F-RCA-I) stained cells (Fig. 2e). In wild type CHO-S cells, the majority of cells (> 94%) were stained with green fluorescent F-RCA-I (Supplementary Fig. 2a). Likewise, the stable pools, only transfected with donor plasmid, showed a complete shift from green fluorescence negative (Q3 + Q4) to green fluorescence positive population (Q1 + Q2) upon F-RCA-I staining, indicating that the majority of cells have functional MGAT1 as expected. On the other hand, the stable pools transfected with Cas9, sgRNA, and donor plasmid revealed a fraction of 4.6–12.5% of green fluorescence negative cells regardless of mCherry expression from donor plasmid, generating red fluorescence. It demonstrated the presence of cells with knockout of the *Mgat1* gene (Fig. 2e). Next, 5′/3′ junction PCR analysis was applied to evaluate the percentage of target specific knock-ins following isolation of G418-resistant and mCherry positive/ZsGreen1-DR negative clones. In contrast to the high efficiency observed in *COSMC* locus, we did not obtain junction PCR positive clones, indicating very low efficiency of targeted integration in the *Mgat1* locus (Table 1). It is likely that most cells belonging to red fluorescence positive/green fluorescence negative (Q4) population upon F-RCA-I staining correspond to mCherry expressors with NHEJ mutation in *Mgat1* locus, and not the targeted integrants. Hence, we used RCA-I lectin selection in order to enrich for *Mgat1*-disrupted clones occurred by targeted integration. RCA-I treatment effectively induced cell growth arrest and subsequent cell death in wild type CHO-S cells, implying RCA-I induced cytotoxicity (Supplementary Fig. 2b). 7 days post-RCA-I treatment, RCA-I resistant cells grew to confluency only in the stable pools transfected with Cas9, sgRNA, and donor plasmid (Supplementary Fig. 2c). F-RCA-I staining of RCA-I enriched cell pools resulted in the major fraction of green fluorescence negative cells preserved in the population of Q3 + Q4, indicating efficient enrichment for the *Mgat1*-disrupted population (Fig. 2e). Deep sequencing of RCA-I enriched pools confirmed this observation with indel frequencies of 83.5% and 99.0% for the sgRNA1 and sgRNA5 target sites, respectively (Supplementary Fig. 2d). Thus, lectin selection can be applied to increase the frequency of *Mgat1*-disrupted clones. RCA-I resistant clones were then isolated from two RCA-I resistant stable cell pools in order to analyze *Mgat1* targeted integrants. 5′/3′ junction PCR analysis of clonal cells revealed the presence of 32.3% and 27.1% PCR positive clones for sgRNA1 and sgRNA5 target sites, respectively (Table 1). Out-out PCR specific for two *Mgat1* integration sites verified the correct targeting of target integration unit accompanying the expected size of amplicons (wild type amplicon: 1.9 kb and 2.0 kb + target integration unit: 3.7 kb ≈ 5.6 kb and 5.7 kb; Percentage of out-out PCR positive clones from 5′/3′ junction PCR positive clones = 90% and 100%, i.e. 29.0% and 27.1% in total; Fig. 2f, Lane 4 and 8, Table 1). As a result, targeting efficiencies of 10.2% and 16.4% for the sgRNA1 and sgRNA5 target sites respectively, were obtained (Table 1). As observed in *COSMC* locus, *Mgat1* locus was also completely disrupted in most targeted clonal cells (20 out of 25 clones), which showed a relative *Mgat1* copy number of close to 0 compared with the *Mgat1* in CHO-S wild type cells (Fig. 2g, left panel). The mCherry gene dosage was found to be consistent amongst clones except three clones, as shown by relative quantification of mCherry gene (Fig. 2g, right panel). Precise targeted integration at the *Mgat1* locus determined that this platform was not restricted to the *COSMC* locus and/or *COSMC*-targeting sgRNA.

### Application of targeted integration platform to the *LdhA* locus

**Figure 3:**
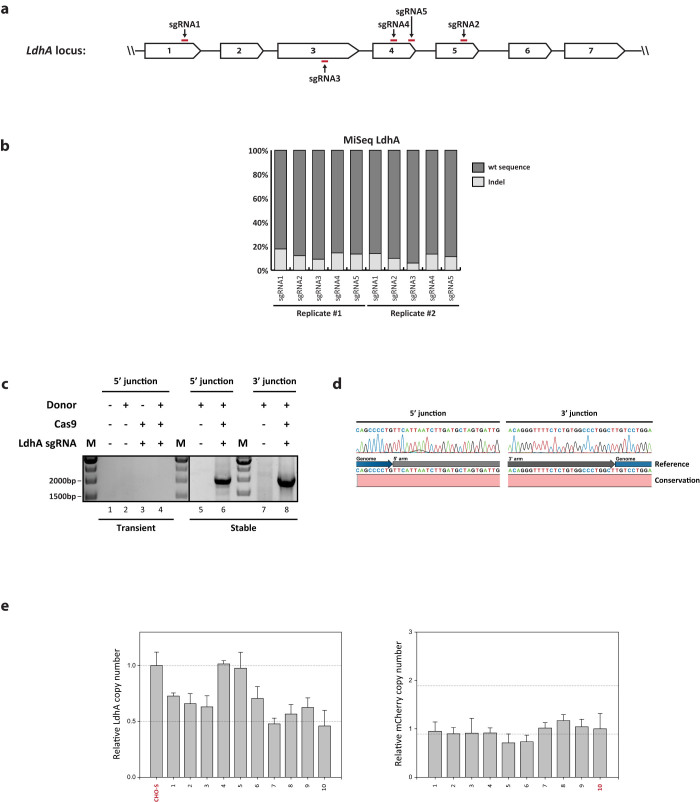
Targeted integration into *LdhA* locus using CRISPR/Cas9. (a) Illustration of the five sgRNA target genomic sites in *LdhA* locus. (b) Indel frequency in *LdhA* locus analyzed by deep sequencing. Genomic DNA was extracted 3 days after transfection with plasmids expressing Cas9 gene and sgRNA. The genomic regions covering sgRNA target sites were amplified, then subjected to deep sequencing analysis using Miseq. The percentage of wt and indel sequences are described in the bar plot. The values from control samples transfected only with plasmid expressing Cas9 were subtracted from test samples. Investigation of target specific knock-in in transiently transfected and stable cell pools analyzed by (c) Agarose gel of 5′/3′ junction PCR (d) Sanger sequencing of the 5′/3′ junction PCR amplicons. Amplicons from the stable cell pool were purified and directly sequenced after PCR amplification. M, 1 kb DNA ladder. (e) Relative copy number of *LdhA* and *mCherry* regions in clonal cells, as described in Fig. 1(e).

We also tested the current integration strategy on *LdhA* locus. *LdhA* encodes the A subunit of lactate dehydrogenase that is responsible for interconversion of pyruvate and lactate, one of the main accumulated waste products during CHO cell culture. *LdhA* is therefore a promising metabolic target gene for knockout in order to enhance culture performance. Five sgRNAs were designed for targeting exon 1, exon 3, exon 4, and exon 5 of the *LdhA* locus (Fig. 3a), followed by deep sequencing analysis to compare the indel efficiency (Fig. 3b). Indel frequencies were reported from 7.6% to 15.9%. Based on this, sgRNA2 target site was selected for homology arm design and integration site. Comparable to the previous loci tested, co-transfection of the donor plasmid with Cas9 and sgRNA plasmid resulted in precise targeted integration of donor DNA into the *LdhA* locus. It was only observed in the stable selected pool as determined by junction PCR analysis with expected DNA bases at the genome-donor boundaries (Fig. 3c, Lane 6 and 8, Fig. 3d). Amongst 61 isolated clonal cells, 10 clones were identified as both junction PCR positive (16.4%, resulting in targeting efficiency of 7.4%, Table 1). In contrast to relative copy number of *COSMC* and *Mgat1* locus, most *LdhA* targeted clonal cells (eight out of 10 clones) yielded a partial number of relative *LdhA* copy number, indicating heterozygous disruptions at the *LdhA* locus caused by monoallelic knock-ins (Fig. 3e, left panel). Unexpectedly, two clones (clone #4 and #5) had unmodified copy numbers of *LdhA* relative to CHO-S wild type cells, probably due to non-clonal cell population or chromosomal instability. Heterozygous modification at *LdhA* locus led to decrease in mRNA expression levels of *LdhA* by approximately 50%, resulting in reduction of protein expression levels as well (Supplementary Fig. 3). Notably, deep sequencing analysis of nonintegrated allele revealed evidence of in-frame deletions created in *LdhA* targeted clonal cells, which is unlikely to lead to a loss-of-function (Supplementary Fig. 4). As a result of heterozygous modification in *LdhA* locus, we could not obtain expected size of PCR products from out-out PCR due to existence of a residual allele. From analysis of the relative mCherry copy number, similar results of consistent copy number across *LdhA* target clonal cells were obtained (Fig. 3e, right panel). Targeted integration at the *LdhA* locus was achieved using the current platform, which confirmed that this method was also applicable at a diploid locus.

### Off-target effects associated with CRISPR/Cas9 mediated targeted integration

**Figure 4:**
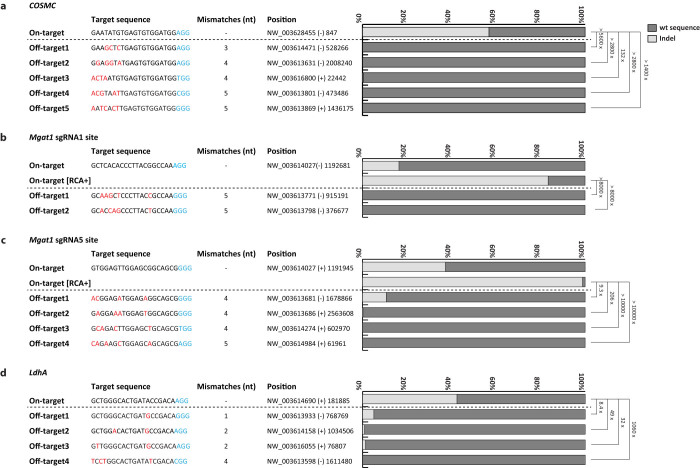
Comparison of mutation rates at on-target and potential off-target sites for (a) *COSMC* sgRNA2, (b) *Mgat1* sgRNA1, (c) *Mgat1* sgRNA5, and (d) *LdhA* sgRNA2. Information on position of sequences consists of contig, strand, and position within the corresponding contig. Mismatched bases and PAM sequences are shown in red and blue, respectively. Genomic DNA extracted from stable cell pools transfected with Cas9, sgRNA, and donor plasmid was used as template for PCR. PCR amplicons spanning corresponding on-target sites and potential off-target sites were subjected to deep sequencing analysis using Miseq. Indel frequencies at off-target sites of *Mgat1* sgRNA1/sgRNA5 site were based on genomic DNA extracted from RCA-I resistant cell pools. Fold differences in indel frequencies between on-target and off-target sites were presented next to the bar plot.

To assess potential off-target mutations introduced by the CRISPR/Cas9 activity, we first identified the most probable potential off-target sites of each sgRNA target sequence within the CHO genome. Prediction of off-target sites was based on three criteria (see Methods section), followed by analysis of the NHEJ-mediated indel frequencies at the on-target and off-target sites in genomic DNA isolated from stable cell pools transfected with Cas9, sgRNA, and donor plasmid by deep sequencing (Fig. 4). A low level (< 0.5%) of off-target mutations were detected at all five putative off-target sites for the *COSMC* sgRNA, despite high levels of CRISPR/Cas9 activity at the corresponding on-target site, which generated indels at frequencies of 56.8% (Fig. 4a). A similar off-target tendency was observed for the *Mgat1* sgRNA1, but unlike sgRNA1, *Mgat1* sgRNA5 displayed off-target indel generation at a frequency of 10.7% at off-target 1 (Fig. 4b,c). RCA-I enrichment for *Mgat1*-disrupted cells drastically increased an indel frequency from 16.4% and 37.3% to 83.4% and 98.9% at sgRNA1 and sgRNA5 on-target sites (Fig. 4b,c), but did not induce additional off-target indel mutations (Supplementary Fig. 5). Three out of four off-target sites for the *LdhA* sgRNA displayed off-target activity (Fig. 4d). Sites harboring one- or two-base mismatches contained indel mutations at a frequency between 0.9% and 5.1%. Comparing the indel frequencies observed at the off-target sites with those at the on-target site, all on-target frequencies were 8.4-fold higher as a minimum. Upon enrichment for positive integrant pools by RCA-I selection, on-target to off-target frequency difference was greatly increased from 9.3-fold to >10,000-fold (Fig. 4b,c). Due to these on-target to off-target frequency differences, integration events at the potential off-target sites will be less likely to occur. PCR amplification of regions, covering off-target sites in mCherry positive/ZsGreen1-DR negative targeted clones, showed no off-target integrations, which supports this hypothesis (Supplementary Fig. 6). The low mutation level of overall off-target sites supported the reliability of the use of CRISPR/Cas9 system for targeted integration.

### Correlation between targeted integration and homogeneous expression of GOI

**Figure 5:**
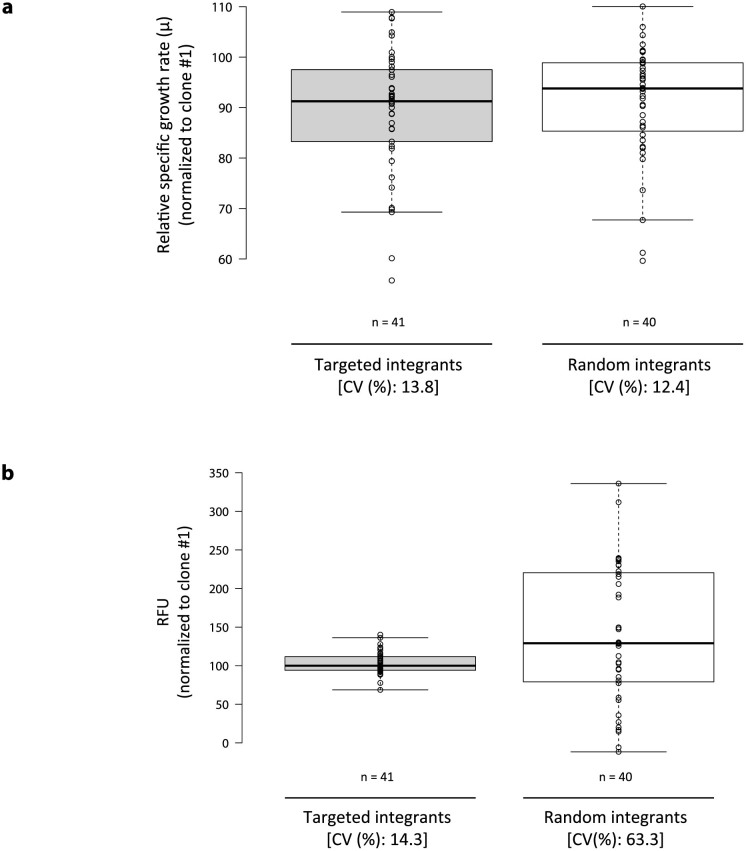
Effect of targeted integration on homogeneous expression of GOI. Targeted and random integrants isolated from the stable cell pool transfected with Cas9, sgRNA, and donor plasmid targeting *COSMC* locus were cultivated to assess (a) RFU and (b) relative specific growth rate (µ). Average mean intensity of mCherry fluorescence measured by imaging cell cytometer was used for RFU. The specific growth rate was calculated from a plot of viable cell concentration against culture time from day 0 to day 3. Each measured value was normalized by that of the clone number 1. Center lines show the medians; box limits indicate the 25th and 75th percentiles as determined by R software; whiskers extend 1.5 times the interquartile range from the 25th and 75th percentiles, outliers are represented by dots; data points are plotted as open circles. n = 41, 40 sample points. Each sample point represents average value of measurements at 3–5 different passages during cultivation for a month. (CV) coefficient of variation.

To assess the effect of targeted integration on cell culture performance, differences in expression of mCherry and growth rates between targeted integrants and random integrants were analyzed. mCherry positive/ZsGreen1-DR negative cell population was isolated from stable cell pool transfected with Cas9, sgRNA2, and donor plasmid targeting *COSMC* locus by limiting dilution, then classified as either targeted integrants or random integrants according to results from 5′/3′ junction PCR and out-out PCR (Supplementary Fig. 7). Both 5′/3′ junction PCR and out-out PCR positive clones were defined as targeted integrants, and out-out PCR negative clones were regarded as random integrants regardless of 5′/3′ junction PCR results. Amongst positive targeted integrants, clones with complete disruption at the *COSMC* locus and similar levels of relative mCherry copy number were selected (Fig. 1e, n = 41). Cell growth and expression level of GOI of the two populations was then compared. Both populations displayed similar variations in the relative specific growth rate (CV: 13.8% versus 12.4%; Fig. 5a). An average expression level of targeted integrants was slightly lower than that of random integrants (RFU average: 103 versus 140, *p* < 0.05; Fig. 5b). Interestingly, targeted integrants displayed more stable mCherry expression with 4.4-fold less variation than that of the random integrants (CV: 14.3% versus 63.3%; Fig. 5b), indicating more homogeneous and predictive expressions from site-specific integration clones. The variability in expression levels of random integrants was caused by the influence of both copy number and integration site (Supplementary Fig. 8). Some of random integrants with 2- or 3-fold higher copy number exhibited 2- or 3-fold higher expression levels relative to the representative targeted integration clone. On the other hand, various expression levels were observed in a few random integrants despite similar levels of copy number. Moreover, although the percentage of mCherry positive/ZsGreen1-DR negative cells was initially close to 100% in all clones investigated, it was notably decreased in some clones (five out of 40 clones, i.e. 12.5% in total) only from the population of random integrants while cells were expanding, underscoring unstable expression from random integration (Supplementary Fig. 9). These data show that CRISPR and HDR mediated targeted integration allowed stable and reproducible transgene expression in rCHO cell lines without affecting cell growth.

## Discussion

Site-specific integration of large transgenes during rCHO cell line construction has been impeded by extremely low integration efficiencies and specific requirements, including construction of platform cell lines, which limit flexible targeting to any desired sites. Here we developed a method for targeted integration by combining the CRISPR/Cas9 system with a donor plasmid in CHO cells. The procedure allowed precise HDR-mediated integration of large gene expression cassettes into desired sites with a targeting efficiency between 7.4–27.8% depending on target locus and CRISPR/Cas9 activity. With the previous targeted integration studies in CHO cells relying on the NHEJ pathway for integration of short (< 100 bp) oligonucleotides^16^ or linear transgenes^17^, our approach is liberated from the issues concerning orientation of transgene integration and imperfect ligation caused by degradation of donor ends by exonuclease activity or end resection of the chromosome. Furthermore, we report additional benefits common to the CRISPR/Cas9 system such as high efficiency, robustness, simplicity, ease of design, and low cost.

Compared with the successful HDR-mediated integration of transgenes in human cells using the CRISPR/Cas9 system, which was observed 72 hours after transfection^14^, we did not detect integration events in transiently expressed cells, but only in stable selected pools although transgene size and experimental conditions were different (Fig. 1b, 2c, and 3c). This result supports intrinsically lower levels of HDR-based transgenesis in CHO cells, which agrees with previous studies^16^,^17^. In particular, the Mgat1 locus had non-appreciable frequencies of integration before RCA-I enrichment, which showed the locus-to-locus variation in terms of integration frequency (Table 1). Although the CRISPR/Cas9 system generated indel mutations via NHEJ with high efficiency between 16.4–56.8%, the rate of HDR-mediated integration remained relative low (Fig. 4, Table 1). The present targeting efficiency is sufficient for the generation of rCHO cells when paired with drug or phenotypic selection, but requirements for selection process can limit the practical application of targeted integration at certain loci in an actual industrial production process. However, the current method has sufficient room for further improvement in targeting efficiency by implementing recent advancements, such as rational design of highly active sgRNAs^23^, enrichment of cells with genome editing events^24^, or construction of expression vectors bearing both Cas9 and the sgRNA^11^ since the CRISPR/Cas9 system is continuously undergoing optimizations to improve activity. Transfection efficiency, which was measured based on the fraction of mCherry positive/ZsGreen1-DR positive cells 72 hours post transfection, was in the range of 24.4–34.0% when three plasmids, including donor plasmid, Cas9, and sgRNA plasmid, were co-transfected. Compared with transfection efficiency of 33.0–54.6% for control samples transfected with the donor plasmid only, transfection of multiple plasmids lowered the transfection efficiency considerably. Additionally, expression levels from separate constructs may not be tightly coupled. This potential limitation can be overcome by using the aforementioned single expression vector^11^ to drive both Cas9 and sgRNA or FACS enrichment to aid selection of transfected cells. As observed in the on-target indel efficiency in stable cells and the targeting efficiency, the higher on-target indel efficiency is likely to lead to higher HDR-mediated integration (Fig. 4, Table 1). Therefore, the aforementioned approaches will aid in the increase in current targeting efficiencies. Moreover, single-strand breaks (SSBs) created by single sgRNA-Cas9 nickases have reported to be capable of inducing homologous recombination in mammalian cells, albeit at reduced frequency compared with wild-type Cas9^25^,^26^. In conjunction with improvements in targeting efficiency, targeted integration via SSBs-induced homologous recombination will also be an attractive strategy with regard to higher specificity and fidelity.

Previous studies have reported that the PAM-proximal 8–13 bp of the target region is crucial for target recognition and cleavage by Cas9^11^,^19^, and within PAM-proximal region, a single-base mismatch can be acceptable to conserve activity. Furthermore, up to three to five mismatches in the PAM-distal region can be tolerated without impairing the ability to cleave DNA^27^,^28^,^29^. More recently, genome-wide mapping of Cas9 binding sites using catalytically inactive Cas9 and ChIP-seq analysis has revealed that a 5-nucleotide seed region next to PAM is required for Cas9 binding in vivo and in vitro^30^. Based on this, we selected potential off-target sites for each target site to meet these criteria, and evaluated off-target mutation rates (Fig. 4). Nine out of 15 potential off-target sites were not affected. In four off-target sites, we detected indels with a frequency of 0.5–1.3%, which was rare enough to allow high fidelity of sgRNAs targeting on-target sites. The last two off-target sites, however, contained detectable off-target cuttings at a frequency of 5.1% and 10.7%. A recent paper has suggested that in addition to finding off-targets at sites with few mismatches compared with the target sequence, off-targets may also be found at sites with small inserts/deletions compared with the target sequence under certain circumstances^31^. This type of off-targets have not been considered in the current work, but guide RNA design in the future should aim to identify such additional potential off-target sites so that appropriate care may be taken. Off-target mutations may not be critical to rCHO cell line construction compared with human gene therapy. However, potential problems in connection with off-target mutations could cause unpredictable toxic effects. This latent obstacle could also be mitigated by using approaches to improve on-target specificity of Cas9, such as the double-nicking approach^26^,^29^, truncated guide RNAs^32^, or dimeric CRISPR RNA-guided FokI nucleases^33^.

Cultivation of targeted integrants displayed more stable and highly reproducible expression levels when compared with random integrants. Although this result demonstrates the minimal variability in expression level, it is limited to the specific locus and expression cassette. The expression level can depend on the target locus and the cassette design, particularly relating to the promoter used and its susceptibility to silencing. Thus, additional studies in various loci with different transgene expression cassettes are warranted to confirm the generality of this finding. Despite homogeneous transgene expression levels in targeted integrants, some of the random integration clones exhibited 2- or 3-fold higher expression levels relative to representative targeted integration clone (Fig. 5b, Supplementary Fig. 8). These observations were due to random integration into highly active chromosomal regions with the CHO genome or integration of several copies of the donor plasmid. Given that the ultimate goal for construction of production cell lines is rapid generation of high and stable producers, ensuring comparable quality for regulatory approval, the selection of ideal transgene insertion sites is highly demanded. Ideal insertion sites, also called ‘Safe harbors’ or ‘Hot spots’, should allow high and stable transcription/expression levels of the GOI without perturbing essential regulatory elements or disrupting genes which are responsible for the quality of the products. Previous extensive studies on the selection and identification of desirable target sites in human cells provide reliable guidelines to facilitate rapid identification of such “hot spots” in CHO cells^13^,^34^. A further refinement of existing Chinese hamster reference genome and all of the relevant sequence information of CHO host cell lines, together with new data on genome stability at the chromosomal level will facilitate identification of potential target sites as well as a better characterization of the current targeted integration strategy. Moreover, a recent study demonstrated that actively transcribed chromatin structure was preferentially repaired by homologous recombination over NHEJ upon DSB^35^. This trait fits perfectly into the presented platform and will reinforce the targeting efficiency. Thus, this approach should be able to provide a valuable genetic tool to characterize different potential good integration sites in CHO cells, ultimately leading to a rational construction of production cell lines assuring sustainable and high expression levels.

## Methods

### Plasmid design and construction

The CHO codon optimized Cas9 expression vector and sgRNA expression vector applied in this study were constructed as described previously^20^. The CRISPy bioinformatics tool^20^ was applied for generating sgRNA target sequences (Supplementary Table 1). Subsequently, single stranded oligos comprising the variable region of the sgRNA (Supplementary Table 2) were synthesized, annealed, and mixed with the expression vector backbone harboring U6 promoter, scaffold RNA sequence, and termination sequence to generate sgRNA expression vectors.

Donor plasmids were constructed with uracil-specific excision reagent (USER) cloning method according to Ref. 36. The different parts of the plasmid were amplified with uracil-containing primers and proofreading polymerase *Pfu*X7. 5′ and 3′ homology arms flanking each sgRNA target sequence were amplified from genomic DNA of CHO-S cells, which were extracted by GeneJET Genomic DNA Purification Kit (Thermo Fisher Scientific, Waltham, MA). EF-1α promoter, NeoR expression cassette (pSV40-NeoR–SV40 pA), and expression vector backbone parts were directly amplified from commercial expression vectors, but ZsGreen1-DR expression cassette (pCMV-ZsGreen1-DR-BGH pA) and mCherry-BGH pA parts were amplified from pre-assembled expression vectors that were constructed with USER cloning method using commercial expression vectors. Primer sequences and all plasmids used as PCR templates are listed in Supplementary Table 2 and 3, respectively. Following purification of PCR products by agarose gel separation and NucleoSpin® Gel and PCR Clean-up kit (Macherey-Nagel, Düren, Germany), the purified 7 PCR fragments including both homology arms, EF-1α promoter, mCherry-BGH pA, NeoR expression cassette, ZsGreen1-DR expression cassette, and backbone were assembled with USER enzyme (New England Biolabs, Ipswich, MA) and transformed into *E. coli* Mach1 competent cells (Life Technologies, Leuven, Belgium). Plasmids were harvested from transformants selected on LB agar plates with ampicillin. All constructs were verified with sequencing and purified using NucleoBond Xtra Midi EF (Macherey-Nagel) according to manufacturer's instructions.

### Cell culture, transfection, and stable cell line construction

CHO-S cells from Life Technologies were grown in CD CHO medium supplemented with 8 mM L-Glutamine (Life Technologies) and cultivated in a 125-mL Erlenmeyer flask (Sigma-Aldrich, St. Louis, MO) with a working volume of 30 mL. The cells were incubated at 37°C, 5% CO_2_ with 120 rpm shaking and passaged every 3 days. Cells were transfected with expression vectors encoding CHO codon optimized Cas9, sgRNA targeting the integration site and corresponding donor plasmid. For each sample, 3 × 10^6^ cells were transfected with a total of 3.75 µg of DNA using FreeStyle^TM^ MAX reagent together with OptiPRO SFM medium (Life Technologies) according to manufacturer's recommendations. 16 hours post transfection, the samples were incubated at 30°C for 32 hours before transferred back to 37°C.

Stable cell pools were generated by seeding cells in CELLSTAR® 6 well Advanced TC plates (Greiner Bio-one, Frickenhausen, Germany) on day 3 followed by selection process under G418 (500 μg/mL; Sigma-Aldrich). During selection, medium was changed every 3–4 days. After 2 weeks of selection, cells were detached with TrypLE (Life Technologies) and adapted to grow in suspension in non-tissue treated plates or Erlenmeyer flask depending on cell concentrations. For clonal selection, limiting dilution step was followed using the stable cell pools, which were either bulk sorted for mCherry positive/ZsGreen1-DR negative cell population using a BD FACSJazz cell sorter (BD Biosciences, San Jose, CA) or not. 1 cell was seeded per well in 200 ul medium in 96 well plates. The generated colonies were analyzed by a Celigo Imaging Cell Cytometer (Nexcelom Bioscience, Lawrence, MA) applying the Direct cell counting application to check for single colonies. Only the wells with round shaped colonies expressing mCherry were selected for further analysis.

### Genomic DNA extraction and PCR amplification

Genomic DNA was extracted from the cell pellets using QuickExtract DNA extraction solution (Epicentre, Illumina, Madison, WI) or GeneJET Genomic DNA Purification Kit depending on the purpose of experiment. For single cell clones, DNA was isolated by adding 20 µL of QuickExtract DNA extraction solution when cells were confluent in 96 well plates. The mixture was then incubated at 65°C for 15 min, followed by 5 min incubation at 98°C. Of this mixture, 1–2 µL were used as template for junction PCR and out-out PCR, as described below. For the other experiments, GeneJET Genomic DNA Purification Kit was used to extract DNA from cell pellets up to 5 × 10^6^ cells according to manufacturer's instructions. 5′/3′ junction PCR was carried out using DreamTaq DNA polymerase (Thermo Fisher Scientific) by touchdown PCR (95°C for 2 min; 10×: 95°C for 30 s, 68°C–58°C (−1°C/cycle) for 30 s, 72°C for 2 min; 20×: 95°C for 30 s, 58°C for 30 s, 72°C for 2 min; 72°C for 5 min). Out-Out PCR was performed using Phusion Hot Start II High-Fidelity DNA Polymerase (Thermo Fisher Scientific) by touchdown PCR (98°C for 30 s; 20×: 98°C for 10 s, 64°C–54°C (−0.5°C/cycle) for 30 s, 72°C for 3 min; 30×: 98°C for 10 s, 54°C for 30 s, 72°C for 3 min; 72°C for 10 min). PCR primers are listed in Supplementary Table 2.

### Deep sequencing analysis

Deep sequencing was performed on a MiSeq Benchtop Sequencer (Illumina, San Diego, CA). Libraries were prepared based on Illumina ‘16S Metagenomic Sequencing Library Preparation’ with some modifications. Genomic regions flanking target genomic sites were amplified using Phusion Hot Start II HF Pfu polymerase (Thermo Fisher Scientific) by standard PCR (98°C for 30 s; 25×: 98°C for 10 s, 59°C for 30 s, 72°C for 30 s; 72°C for 10 min), with primers containing overhang sequences compatible with Illumina Nextera XT indexing (forward primer overhang: TCGTCGGCAGCGTCAGATGTGTATAAGAGACAG, reverse primer overhang: GTCTCGTGGGCTCGGAGATGTGTATAAGAGACAG). PCR primers are listed in Supplementary Table 2. After PCR amplification, amplicons were purified using AMPure XP beads (Beckman Coulter, Brea, CA). Illumina Nextera XT Index (Illumina # FC-131-1001) sequencing adapters were integrated into the amplicons by PCR (98°C for 3 min; 8×: 95°C for 30 s, 55°C for 30 s, 72°C for 30 s; 72°C for 5 min). The final libraries were purified with AMPure XP beads before quantification. The purified libraries were quantified using the Qubit® dsDNA BR Assay Kit (Life Technologies), and the product size within the library was verified by 2% agarose gel. Sequencing was performed as a 151 paired-end run. Sequence data was analyzed using custom python code that compared the sequence data with the expected wild-type sequence from the CHO genome^20^. In short, it first paired the paired end fastq sequence files. Then, it de-multiplexed the sequence reads based on 5′ or 3′ similarity to the expected wild-type sequence followed by detection of potential indels to categorize sequences as either wild type or indel-containing. The final product of these steps is a list for each target or off-target with numbers for how often we see the wild type and how often we see indels and the size of these indels.

### Batch culture

Exponentially growing cells were inoculated at a concentration of 3.0–4.0 × 10^5^ cells/mL into Polystyrene transparent square 96-half-deepwell microplates with Sandwich cover (Enzyscreen, Leiden, Netherlands) containing 250–300 µL of culture medium, and the culture plates were then incubated on the Multitron cell (INFORS HT, Bottmingen, Switzerland) at 350 rpm in humidified 5% CO_2_ at 37°C. Viable cell concentration was estimated using PrestoBlue® Cell Viability Reagent (Life Technologies) according to manufacturer's instructions. Briefly, 10 µL of PrestoBlue reagent was directly added to 90 µL of the mixture of cells and media in 96 well plates. The plates were incubated for > 30 minutes at 37°C, and then fluorescence was measured by microplate reader (Synergy Mx, BioTek Instruments, Inc., Winooski, VT). The resulting fluorescence intensity was used to determine viable cell concentration based on the standard curve created with CHO-S cells. On day 3 after a new passage, cells were incubated with NucBlue® Live ReadyProbes® Reagent (Life Technologies) for 20 minutes, and red and green fluorescence was measured by a Celigo Imaging Cell Cytometer using the mask + target 1 + target 2 application. Cells were identified using the blue fluorescence channel as mask, and the red and green fluorescent channels were used as target 1 and target 2, respectively. For cytometer analysis, about 10,000–20,000 cells were used per sample.

### Ricinus Communis Agglutinin-I (RCA-I) selection and phenotypic analysis of Mgat1 knockout cells

Mgat1 knockout cells were enriched in culture medium with 5 μg/mL of RCA-I (Vector Laboratories, Peterborough, UK) using the stable cell pools. After 7 days of selection, cells were adapted to grow in suspension, followed by phenotypic analysis. Cells were stained with NucBlue® Live ReadyProbes® Reagent with and without fluorescein labeled RCA-I (F-RCA-I, Vector Laboratories) at final concentration of 20 µg/ml for 45 min at RT. Cells were then washed three times with fresh culture medium and medium was completely removed. Celigo Imaging Cell Cytometer was used to measure fluorescence using the mask + target 1 + target 2 application as described earlier.

### Off-target prediction

Off-targets were predicted using custom python code that compared the targets with all other potential targets in the CHO genome. Off-targets were then scored based on the following criteria: i) The first 5 bp immediately adjacent to the PAM sequence: cutoff value of 0; ii) The number of mismatches in the first 13 bp: cutoff value of up to 1; iii) The total number of mismatches including number of mismatches in first 13 bp: cutoff value up to 5.

### Quantitative Real Time PCR (qRT-PCR) for copy number analysis

For relative determination of copy number of transgene, qRT-PCR was performed on genomic DNA samples using Brilliant III Ultra-Fast SYBR® Green QPCR Master Mix (Agilent Technologies, Santa Clara, CA) on Mx3005P qPCR System (Agilent Technologies) according to manufacturer's instructions. Reaction mixtures contained SYBR Green QPCR master mix, 250 nM of forward and reverse primers, reference dye, and 20 ng of genomic DNA. Amplification was executed with the following conditions: 95°C for 10 min; 40×: 95°C for 20 s, 60°C for 30 s. The primers were designed using PrimerQuest (Integrated DNA Technologies, Coralville, IA) against transgene encompassing *EF-1α – mCherry*, target genes including *COSMC*, *Mgat1* (sgRNA1 and sgRNA5 target site), and *LdhA*, and reference gene, *Vcl* (Supplementary Table 2). All primers were validated empirically by melting curve analysis and agarose gel electrophoresis. Standard curves generated with 4-fold serial dilutions of genomic DNA samples over 5 grades showed a good linearity (r^2^ > 0.98) and acceptable amplification efficiencies between 90% and 110% for all primer pairs. Each PCR reaction included no template controls in every PCR running, and had 3 replicates with 2 times repetition. Using a delta-delta threshold cycle (ΔΔC_T_) method^37^, relative copy number was estimated with respect to wild type CHO-S cells for target genes and representative clones for mCherry transgene.

## Author Contributions

J.S.L. and H.F.K. designed the experiments and wrote the manuscript. J.S.L., T.B.K., L.E.P. performed the experiments. J.S.L., L.E.P. and H.F.K. analyzed the data. T.B.K. and L.E.P. commented on the manuscript.

## Supplementary Material

Supplementary Information Supplementary Information

## Figures and Tables

**Table 1:** Summary of targeted integration

Target locus	Percentage of mCherry+/ZsGreen1-DR- population^a^ (%)	Total clone number (mCherry+/ZsGreen1-DR-)	5′/3′ Junction PCR positive	Out/Out PCR positive^c^	Targeting efficiency^d^ (%)
***COSMC***	45.7 ± 2.6	115	83	70	27.8
***Mgat1*^b^ (Before RCA-I enrichment)**	62.2 ± 2.7/41.1 ± 1.0	124/37	0/0	N/A	N/A
***Mgat1*^b^ (After RCA-I enrichment)**	35.3 ± 2.1/60.4 ± 6.6	31/59	10/16	9/16	10.2/16.4
***LdhA***	45.2 ± 2.9	61	10	N/A	7.4

^a^The numbers indicate the percentage of mCherry+/ZsGreen1-DR- cell population in each stable cell pool measured by imaging cell cytometer. Average values from three independent experiments ± SD are shown.

^b^Two numbers separated by diagonal lines indicate the results from two sgRNA target sites (sgRNA1/sgRNA5).

^c^Positive indicates expected size of PCR products occurring by targeted integration.

^d^Targeting efficiency was obtained by multiplying the percentage of mCherry+/ZsGreen1-DR- cell population by the percentage of out/out PCR positive clones amongst total mCherry+/ZsGreen1-DR-clones investigated. In case of *LdhA* locus, out/out PCR results were not available. Therefore, 5′/3′ junction PCR results were used instead.
