# Supplementary material for: Site-specific integration in CHO cells mediated by CRISPR/Cas9 and homology-directed DNA repair pathway
Source: Sci Rep. 2015 Feb 25;5:8572. doi: 10.1038/srep08572 (PMC4339809; doi:10.1038/srep08572)
Supplement: Supplementary Information [file srep08572-s1.pdf]

# Supplementary Information

## Site Specific Integration in CHO Cells Mediated by CRISPR/Cas9 and Homology-Directed DNA Repair Pathway

Jae Seong Lee, Thomas Beuchert Kallehauge, Lasse Ebdrup Pedersen, and Helene Faustrup Kildegaard*

The Novo Nordisk Foundation Center for Biosustainability, Technical University of Denmark, 2970 Hørsholm, Denmark

* To whom correspondence should be addressed. Tel: + 45 20 12 46 29; Fax: + 45 45 25 80 01; Email: hef@biosustain.dtu.dk


| Section, figures and tables | Page number |
|---|---|
| Supplementary Figure 1 | 2 |
| Supplementary Figure 2 | 3 |
| Supplementary Figure 3 | 4 |
| Supplementary Figure 4 | 5 |
| Supplementary Figure 5 | 6 |
| Supplementary Figure 6 | 7 |
| Supplementary Figure 7 | 8 |
| Supplementary Figure 8 | 9 |
| Supplementary Figure 9 | 10 |
| Supplementary Table 1 | 11 |
| Supplementary Table 2 | 11 |
| Supplementary Table 3 | 15 |

# Figures

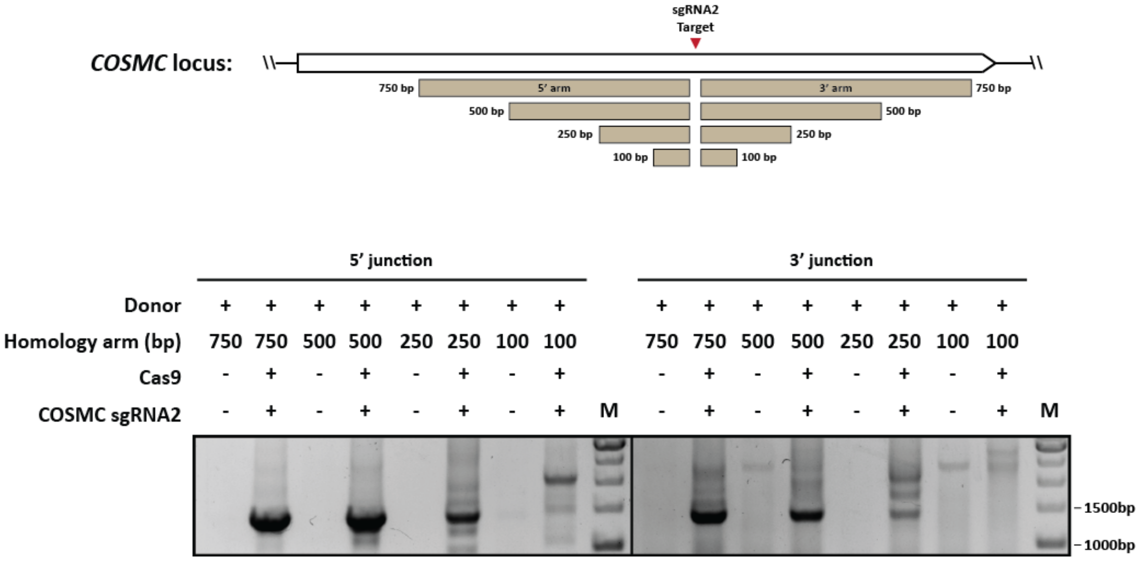


**Supplementary Figure 1. Effect of the length of homology arms on targeted integration.** Donor plasmids containing different length of 5' and 3' homology arms flanking *COSMC* sgRNA2 target site were designed. Genomic DNA extracted from stable pool of cells transfected with Cas9, sgRNA2, and each donor plasmid was used for 5'/3' junction PCR to investigate target specific knock-in.

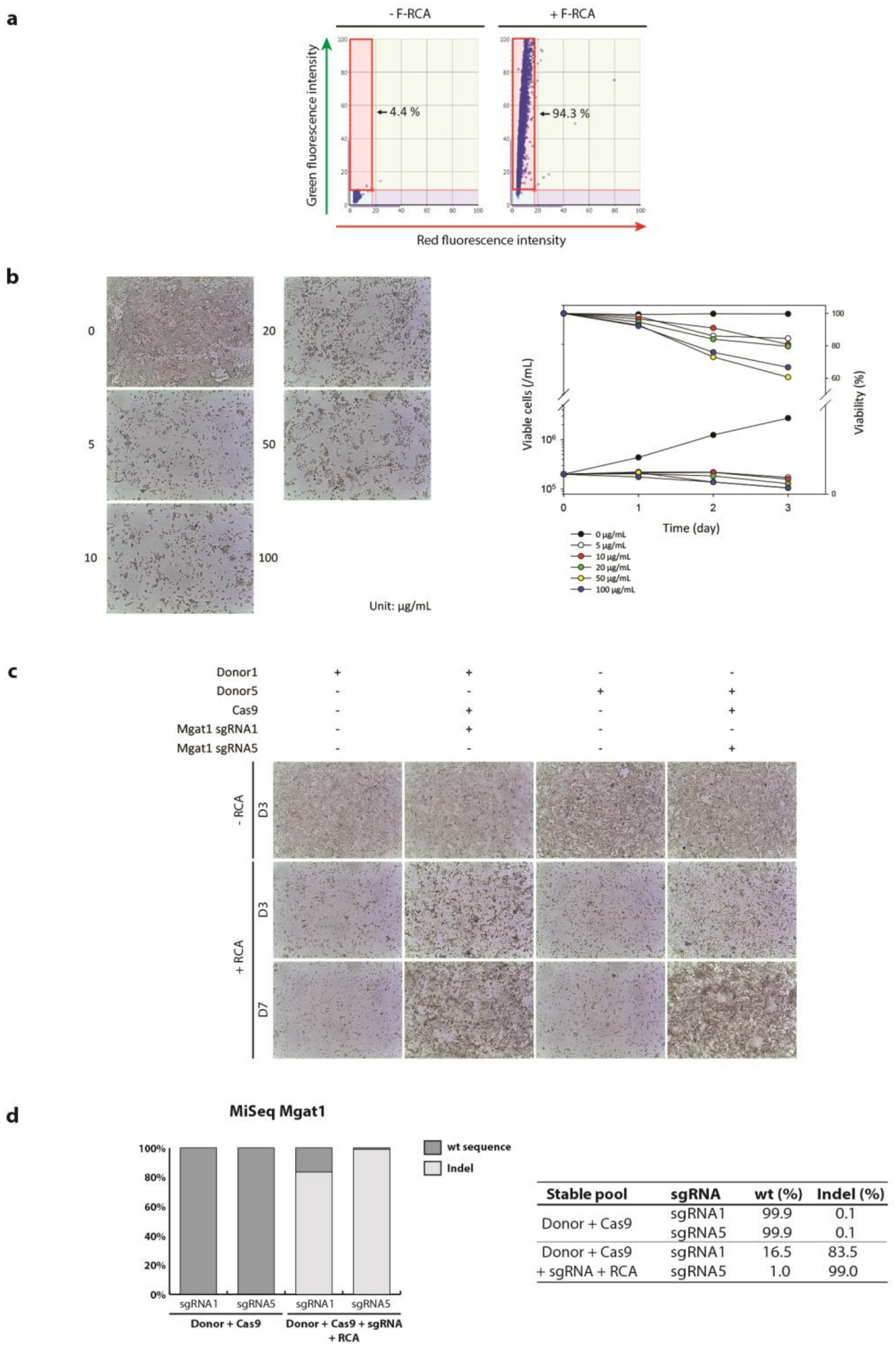


| Stable pool | sgRNA | wt (%) | Indel (%) |
|---|---|---|---|
| Donor + Cas9 | sgRNA1 | 99.9 | 0.1 |
| | sgRNA5 | 99.9 | 0.1 |
| Donor + Cas9 | sgRNA1 | 16.5 | 83.5 |
| + sgRNA + RCA | sgRNA5 | 1.0 | 99.0 |

**Supplementary Figure 2. RCA-I sensitivity analysis on wild type CHO-S cells and *Mgat1* knockout cells.** (a) F-RCA staining of CHO-S cells, followed by quantification based on green fluorescent levels. (b) Effect of RCA-I treatment on CHO-S cells with regard to cell growth and viability. CHO-S cells were cultivated in the presence of various concentrations of RCA-I. Viable cell concentration and viability was subsequently measured on a daily basis, and representative microscopy images were acquired on day 3 (*left*). (c) Acquisition of RCA-I resistance occurred by CRISPR/Cas9 activity for the *Mgat1* locus. Stable pool of cells transfected with Cas9, sgRNA, and donor plasmid targeting *Mgat1* locus formed confluent in the presence of RCA-I. (d) Knockout of *Mgat1* locus in RCA-I resistant cells was confirmed by deep sequencing analysis of the *Mgat1* locus.

**a**

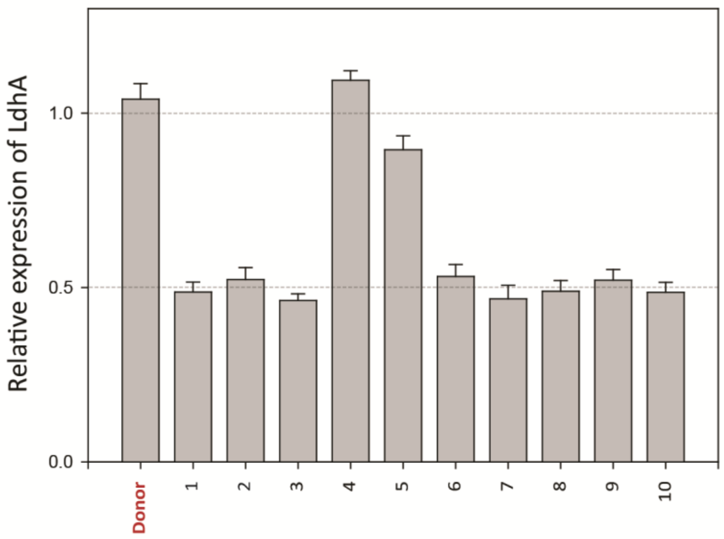


**b**

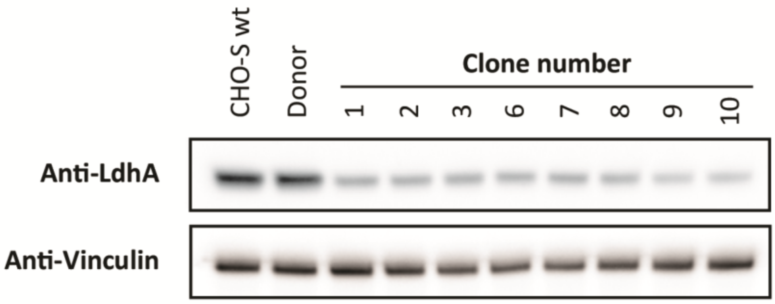


**Supplementary Figure 3. Comparison of the relative LdhA expression level in *LdhA* targeted clonal cells.** (a) Analysis of LdhA mRNA. Total RNAs from each individual clone were isolated using TRIzol® Reagent (Life Technologies), followed by DNase treatment to remove contaminating DNA (TURBO DNA-free™ DNase Treatment and Removal Reagents, Life Technologies). cDNAs were synthesized from 1 µg of total RNAs using Maxima First Strand cDNA Synthesis Kit for RT-qPCR (Thermo Fisher Scientific). For each cDNA sample, the qRT-PCR was performed to quantify LdhA mRNA levels. All samples were normalized for the beta-actin (ACTB) level. The control used to estimate the relative expressions was the mRNA expression in CHO-S wild type cells, which was set at 1.0. Average values from three independent experiments ± SD are shown. (b) Analysis of LdhA protein. Cells were lysed in a fresh lysis buffer (10 mM Tris-HCl, pH 7.4, 100 mM NaCl, 2.5 mM $MgCl_2$, 0.5% Triton X-100, supplemented with 1x Roche protease inhibitor cocktail). 10 µg of protein was separated on 4-12% Bis-Tris NuPAGE® gel (Life Technologies), followed by transfer to nitrocellulose membranes. Blots were probed with anti-LdhA (Cell Signaling Technology, Danvers, MA) or anti-Vinculin (Sigma-Aldrich; loading control) antibodies, and bands were then visualized using Amersham ECL Prime Western Blotting Detection Reagent (GE Healthcare Life Sciences, Buckinghamshire, UK).

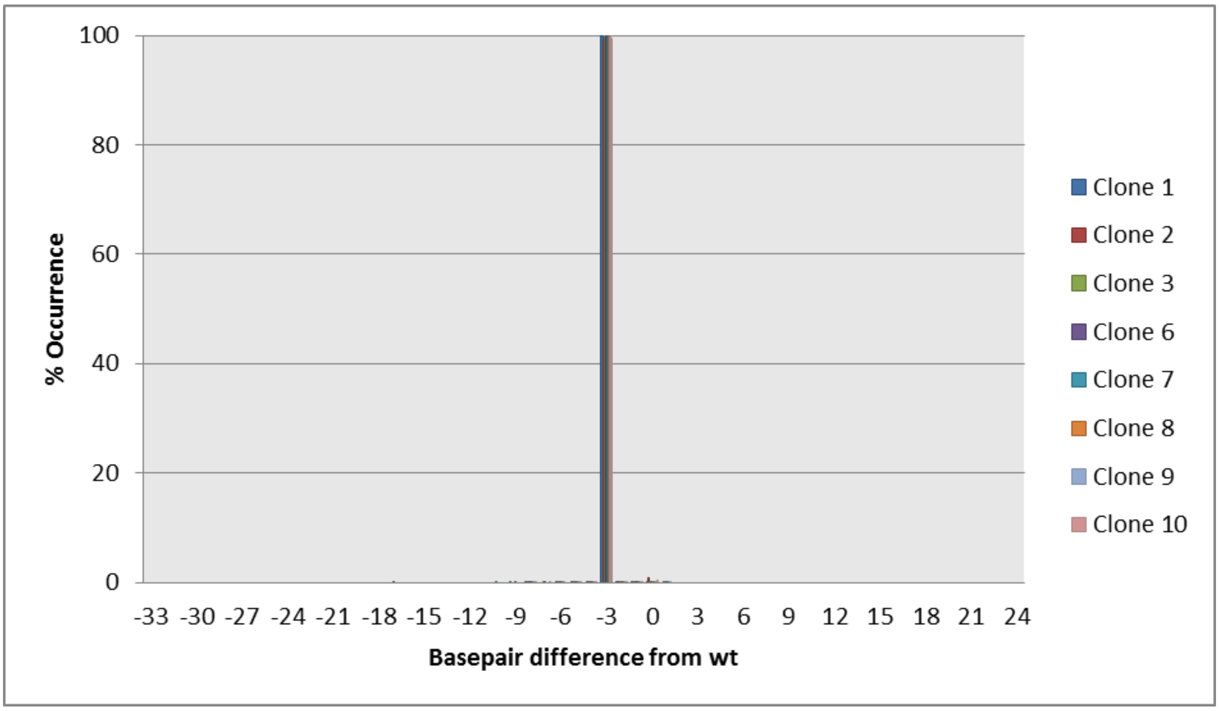


**Supplementary Figure 4. Frequency distribution of indel sizes at the *LdhA* locus in *LdhA* targeted clonal cells.** The regions spanning the integration site at the *LdhA* locus, sgRNA2 target site, were subjected to deep sequencing analysis using Miseq. The size distribution of indels ranging from -33 bp (33 bp deletion) to 24 bp (24 bp insertion) is shown.

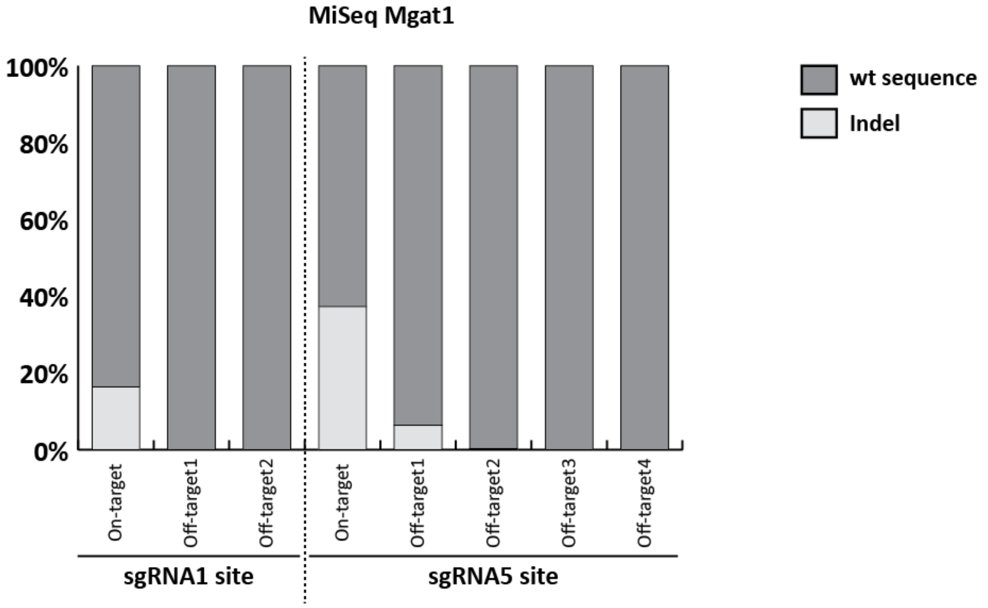


**Supplementary Figure 5. Indel frequencies at *Mgat1* on-target sites and off-target sites before RCA-I enrichment.** Genomic DNA was extracted from two stable pool of cells transfected with Cas9, sgRNA, and donor plasmids, one for each sgRNA (sgRNA1 or sgRNA5). PCR amplicons, which span the *Mgat1* on-target sites and potential off-target sites as described in Fig. 4B and 5C, were subjected to deep sequencing analysis using Miseq.

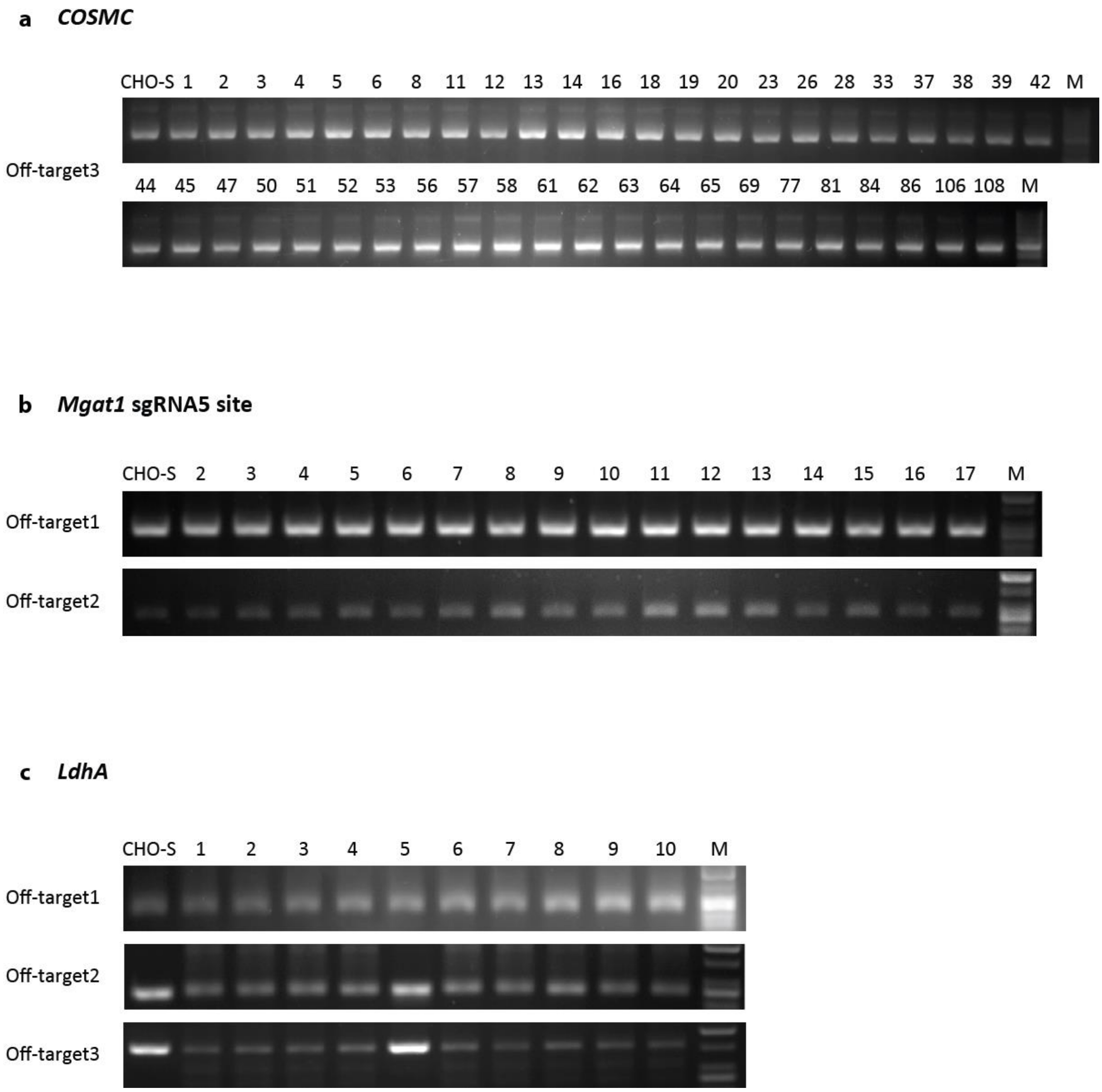


**Supplementary Figure 6. PCR amplification of potential off-target sites**. PCR was performed on genomic DNAs of each individual clone that were used for off-target analysis. PCR amplicons spanning off-target sites at any measurable frequency were generated, and amplicon size was compared with that from CHO-S wild type cells.

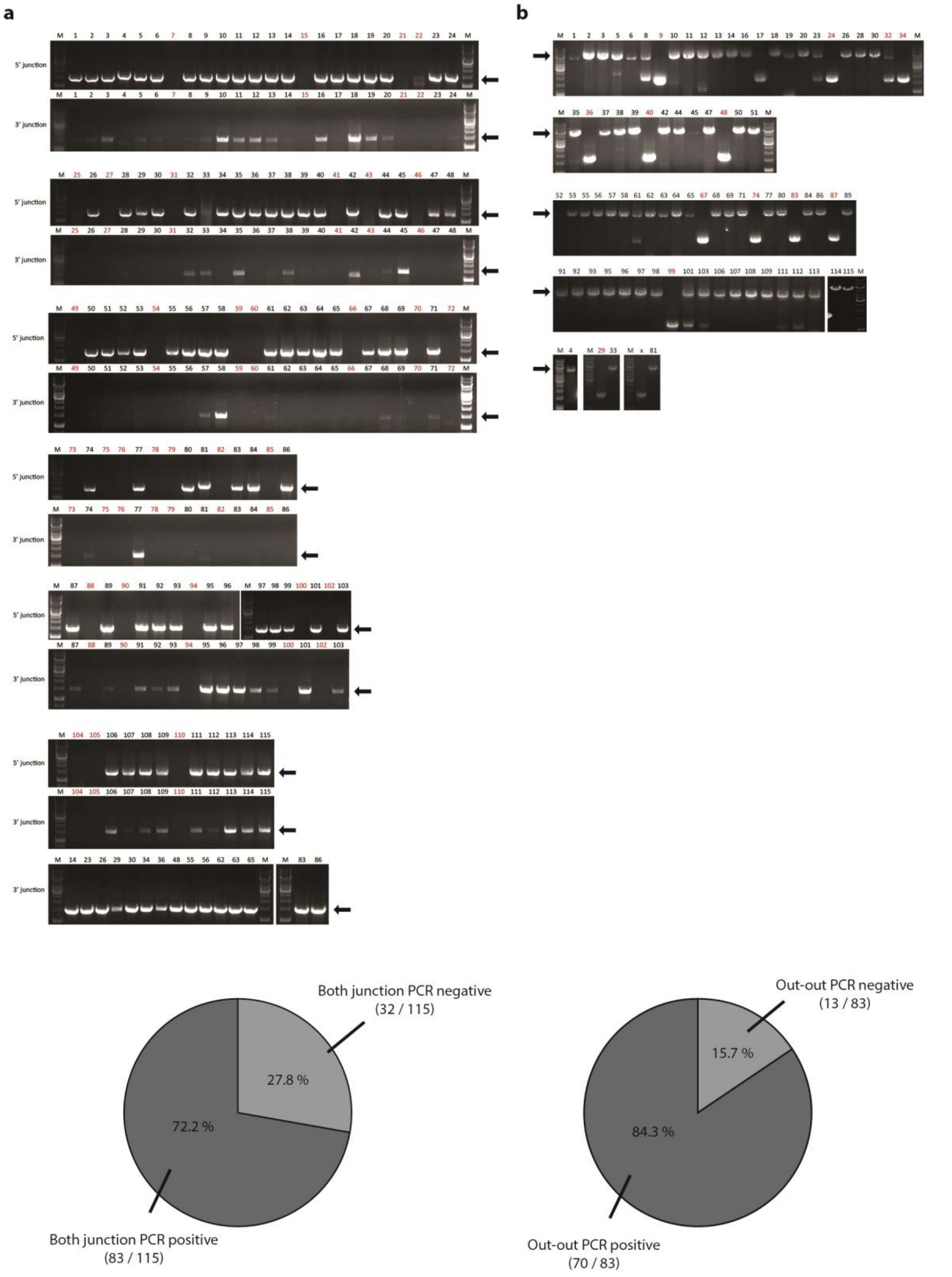


**Supplementary Figure 7. Selection of targeted integrants at the *COSMC* locus.** (a) 5'/3' junction PCR was performed on genomic extract from 115 clones. 83 of the clones (72.2%) were both junction PCR positive, which were further analyzed by (b) out-out PCR. Expected size of amplicons were indicated by arrows. Numerals indicated the number of each clone.

The number of PCR negative clones was shown in red. M, 1 kb DNA ladder. (*bottom*) Pie charts displaying the contribution of PCR positive and negative clones to a total.

**a**

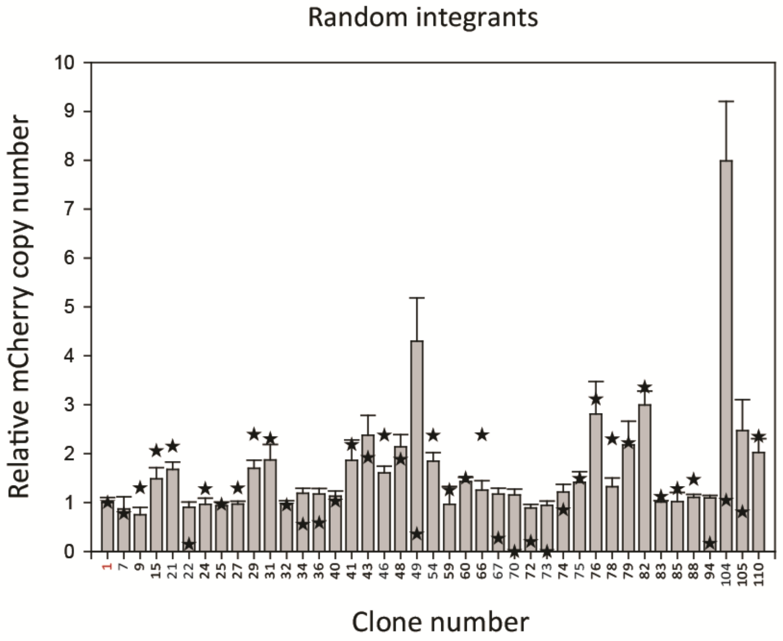


**b**

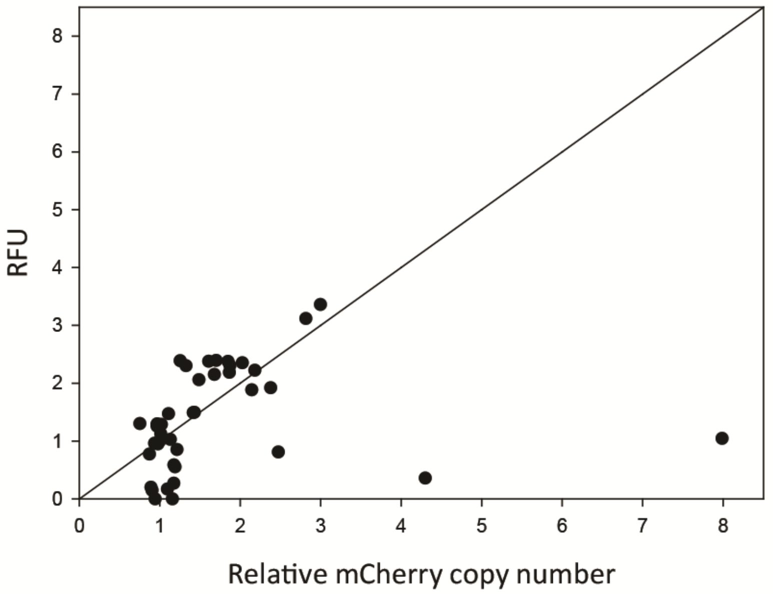


**Supplementary Figure 8. Relationship between the relative copy number of mCherry regions and the expression level of mCherry (RFU) in random integrants.** (a) (Gray bar) relative mCherry copy number; (black star) RFU. Each measured value was normalized by that of the targeted integrant clone number 1 (*COSMC* locus; shown in red). (b) Scatter plot of RFU versus relative mCherry copy number. A diagonal line indicates a one to one correlation.

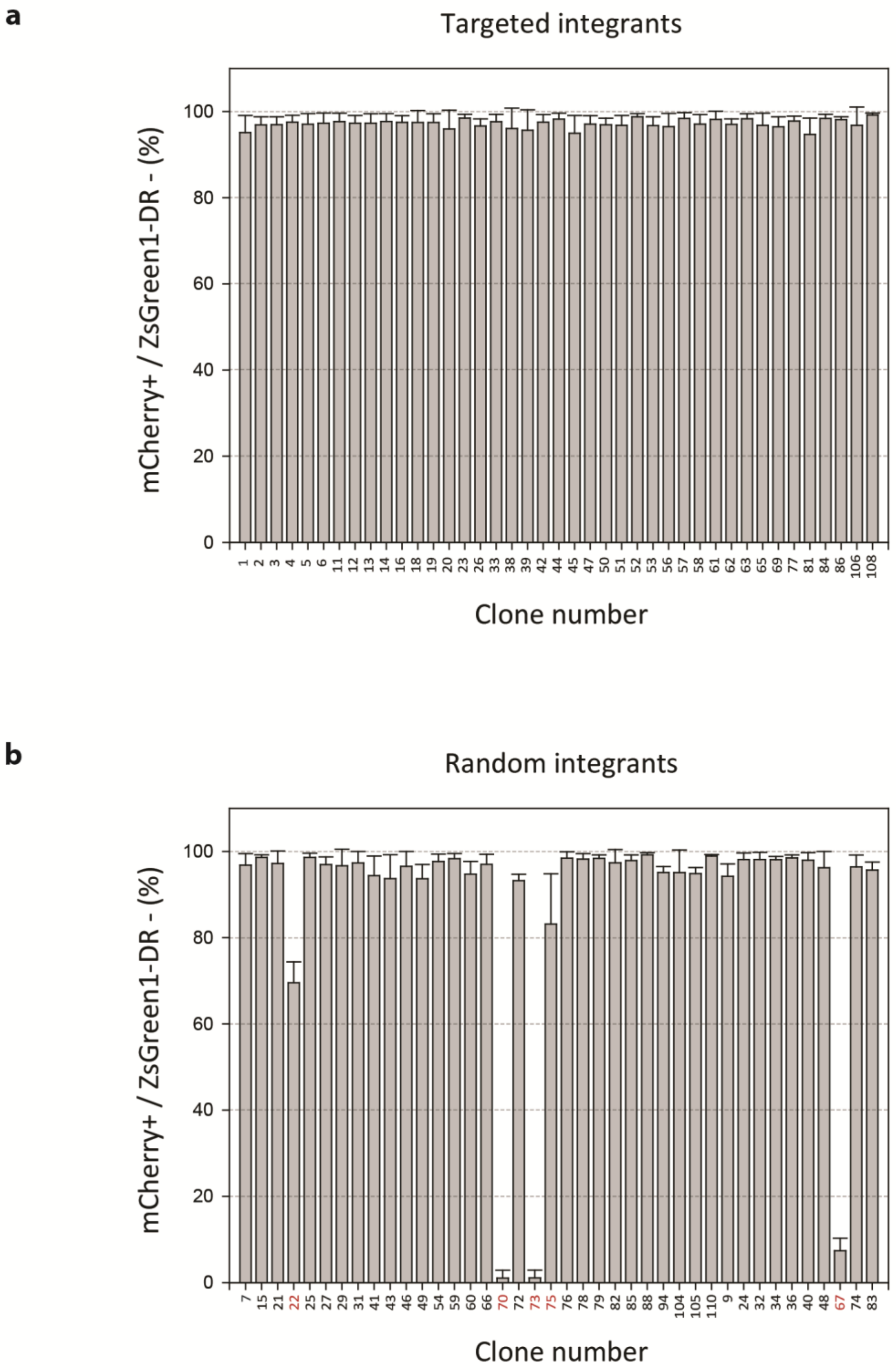


**Supplementary Figure 9. Percentage of mCherry positive/ZsGreen1-DR negative cells in individual clone.** mCherry positive/ZsGreen1-DR negative clonal cells in the population of either (a) targeted integrants (*COSMC* locus) or (b) random integrants were expanded prior to batch cultures and subsequently inoculated for batch cultures. On day 3, the percentage of mCherry positive/ZsGreen1-DR negative cells was measured by imaging cell cytometer. Clones with a decreased percentage were shown in red. The error bars represent the standard deviations calculated from the data obtained in triplicate experiments.

# Tables

**Supplementary Table 1.** sgRNA genomic target sequences

| Target gene | sgRNA name | GNNNNNNNNNNNNNNNNNNNNGG | Ref. |
|---|---|---|---|
| COSMC | sgRNA2_C | GAATATGTGAGTGTGGATGGAGG | Ronda et al. (2014) |
| MGAT1 | sgRNA1 | GCTCACACCCTTACGGCCAAAGG | In this study |
| | sgRNA2 | GGACACGTCGACCCCTGAACTGG | In this study |
| | sgRNA3 | GAGGGGGTCGCAGGCACACGGGG | In this study |
| | sgRNA4 | GGGGCACAGCGATGTTACTCAGG | In this study |
| | sgRNA5 | GTGGAGTTGGAGCGGCAGCGGGG | In this study |
| LDHA | sgRNA1 | GAACAAGATTACGATTGTTGGGG | In this study |
| | sgRNA2 | GCTGGGCACTGATACCGACAAGG | In this study |
| | sgRNA3 | GGACTGTATTTCACAACGTTGGG | In this study |
| | sgRNA4 | GCTCGATTCCGTTATCTGATGGG | In this study |
| | sgRNA5 | GCTGTCATGGATGGGTCCTAGGG | In this study |

**Supplementary Table 2.** Primer sequences

| Primer name | Purpose | Sequence (5'-3') |
|---|---|---|
| | **sgRNA expression plasmid** | |
| Mgat1_sgRNA1_fwd | Sense oligo for Mgat1_sgRNA1 | GGAAAGGACGAAACACCGCTCACACCCTTACG GCCAAGTTTTAGAGCTAGAAAT |
| Mgat1_sgRNA1_rev | Antisense Oligo for Mgat1_sgRNA1 | CTAAAACTTGGCCGTAAGGGTGTGAGCGGTGTT TCGTCCTTTCCACAAGATAT |
| Mgat1_sgRNA2_fwd | Sense oligo for Mgat1_sgRNA2 | GGAAAGGACGAAACACCGGACACGTCGACCCC TGAACGTTTTAGAGCTAGAAAT |
| Mgat1_sgRNA2_rev | Antisense Oligo for Mgat1_sgRNA2 | CTAAAACGTTCAGGGGTCGACGTGTCCGGTGTT TCGTCCTTTCCACAAGATAT |
| Mgat1_sgRNA3_fwd | Sense oligo for Mgat1_sgRNA3 | GGAAAGGACGAAACACCGAGGGGGTCGCAGG CACACGGTTTTAGAGCTAGAAAT |
| Mgat1_sgRNA3_rev | Antisense Oligo for Mgat1_sgRNA3 | CTAAAACCGTGTGCCTGCGACCCCCTCGGTGTT TCGTCCTTTCCACAAGATAT |
| Mgat1_sgRNA4_fwd | Sense oligo for Mgat1_sgRNA4 | GGAAAGGACGAAACACCGGGGCACAGCGATGT TACTCGTTTTAGAGCTAGAAAT |
| Mgat1_sgRNA4_rev | Antisense Oligo for Mgat1_sgRNA4 | CTAAAACGAGTAACATCGCTGTGCCCCGGTGTT TCGTCCTTTCCACAAGATAT |
| Mgat1_sgRNA5_fwd | Sense oligo for Mgat1_sgRNA5 | GGAAAGGACGAAACACCGTGGAGTTGGAGCG GCAGCGGTTTTAGAGCTAGAAAT |
| Mgat1_sgRNA5_rev | Antisense Oligo for Mgat1_sgRNA5 | CTAAAACCGCTGCCGCTCCAACTCCACGGTGTTT CGTCCTTTCCACAAGATAT |
| LdhA_sgRNA1_fwd | Sense oligo for LdhA_sgRNA1 | GGAAAGGACGAAACACCGAACAAGATTACGAT TGTTGGTTTTAGAGCTAGAAAT |
| LdhA_sgRNA1_rev | Antisense Oligo for LdhA_sgRNA1 | CTAAAACCAACAATCGTAATCTTGTTCGGTGTTT CGTCCTTTCCACAAGATAT |
| LdhA_sgRNA2_fwd | Sense oligo for Mgat1_sgRNA2 | GGAAAGGACGAAACACCGCTGGGCACTGATAC CGACAGTTTTAGAGCTAGAAAT |
| LdhA_sgRNA2_rev | Antisense Oligo for LdhA_sgRNA2 | CTAAAACTGTCGGTATCAGTGCCCAGCGGTGTT TCGTCCTTTCCACAAGATAT |
| LdhA_sgRNA3_fwd | Sense oligo for LdhA_sgRNA3 | GGAAAGGACGAAACACCGGACTGTATTTCACA ACGTTGTTTTAGAGCTAGAAAT |
| LdhA_sgRNA3_rev | Antisense Oligo for LdhA_sgRNA3 | CTAAAACAACGTTGTGAAATACAGTCCGGTGTT |

| | | |
|---|---|---|
| | | TCGTCCTTTCCACAAGATAT |
| LdhA_sgRNA4_fwd | Sense oligo for LdhA_sgRNA4 | GGAAAGGACGAAACACCGCTCGATTCCGTTATC TGATGTTTTAGAGCTAGAAAT |
| LdhA_sgRNA4_rev | Antisense Oligo for LdhA_sgRNA4 | CTAAAACATCAGATAACGGAATCGAGCGGTGTT TCGTCCTTTCCACAAGATAT |
| LdhA_sgRNA5_fwd | Sense oligo for LdhA_sgRNA5 | GGAAAGGACGAAACACCGCTGTCATGGATGGG TCCTAGTTTTAGAGCTAGAAAT |
| LdhA_sgRNA5_rev | Antisense Oligo for LdhA_sgRNA5 | CTAAAACTAGGACCCATCCATGACAGCGGTGTT TCGTCCTTTCCACAAGATAT |
| **Donor plasmid** | | |
| COSMC 5' arm_fwd | USER PCR primer for COSMC donor plasmid (Homology arm) | AGTCGGTGUGTAATCCATGGAGGAGTTTCT |
| COSMC 5' arm_rev | USER PCR primer for COSMC donor plasmid (Homology arm) | ACGCTGCTUAAGGTCTCCAGATTTTACAGT |
| COSMC 3' arm_fwd | USER PCR primer for COSMC donor plasmid (Homology arm) | AGGTCTGAGUGATTGTCTTAAGCATAGAGTC |
| COSMC 3' arm_rev | USER PCR primer for COSMC donor plasmid (Homology arm) | AGCGACGUCCTCATTTGCATATATTTGAA |
| Mgat1 5' arm_fwd - set1 | USER PCR primer for Mgat1 donor plasmid (Homology arm) | AGTCGGTGUTTGCAGCAAATCAGGGAGCAT |
| Mgat1 5' arm_rev - set1 | USER PCR primer for Mgat1 donor plasmid (Homology arm) | ACGCTGCTUTCATCGTTCTTGAAATTTCTG |
| Mgat1 3' arm_fwd - set1 | USER PCR primer for Mgat1 donor plasmid (Homology arm) | AGGTCTGAGUCATGGGCAGTTCTTTGATCAGC |
| Mgat1 3' arm_rev - set1 | USER PCR primer for Mgat1 donor plasmid (Homology arm) | AGCGACGUAGGAGCCAGGTTAGGGTCAAC |
| Mgat1 5' arm_fwd - set2 | USER PCR primer for Mgat1 donor plasmid (Homology arm) | AGTCGGTGUTCACTGTGTTTCCTTACTAAGT |
| Mgat1 5' arm_rev - set2 | USER PCR primer for Mgat1 donor plasmid (Homology arm) | ACGCTGCTUCTCAGCGTCCTCAGCCAGG |
| Mgat1 3' arm_fwd - set2 | USER PCR primer for Mgat1 donor plasmid (Homology arm) | AGGTCTGAGUGCTGTTGCAGCAAATCAGGGAG |
| Mgat1 3' arm_rev - set2 | USER PCR primer for Mgat1 donor plasmid (Homology arm) | AGCGACGUCCCTTACGGCCAAAGGTCATCG |
| LdhA 5' arm_fwd | USER PCR primer for LdhA donor plasmid (Homology arm) | AGTCGGTGUTTCATTAATCTTGATGCTAGTG |
| LdhA 5' arm_rev | USER PCR primer for LdhA donor plasmid (Homology arm) | ACGCTGCTUTCTGGATTCAGATTCTTCAGGG |
| LdhA 3' arm_fwd | USER PCR primer for LdhA donor plasmid (Homology arm) | AGGTCTGAGUAGCAGTGGAATGAGGTTCACAA |
| LdhA 3' arm_rev | USER PCR primer for LdhA donor plasmid (Homology arm) | AGCGACGUAGCCAGGGCCACAGAGAAAAC |
| EF-1a_fwd | USER PCR primer for donor plasmid (EF-1a) | AAGCAGCGUGTGAGGCTCCGGTGCCC |
| EF-1a_rev | USER PCR primer for donor plasmid (EF-1a) | ATGACGTCUTCACGACACCTGAAATGGAA |
| kozak_mcherry_fwd | USER PCR primer for donor plasmid (mCherry-BGH pA) | AGACGTCAUCGCCACCATGGTGAGCAAGG |
| BGH pA_rev | USER PCR primer for donor plasmid (mCherry-BGH pA) | ATCGCACUCCATAGAGCCCACCGCATCC |
| Marker NeoR_fwd | USER PCR primer for donor plasmid (NeoR) | AGTGCGAUCTGTGGAATGTGTGTCAGTT |
| Marker NeoR_rev | USER PCR primer for donor plasmid (NeoR) | ACTCAGACCUCAGACATGATAAGATACATTG |
| CMV_fwd | USER PCR primer for donor plasmid (ZsGreen1) | ACGTCGCUGTTGACATTGATTATTGACT |

| | | |
|---|---|---|
| BGH pA_rev2 | USER PCR primer for donor plasmid (ZsGreen1) | ACGCAAGUCCATAGAGCCCACCGCATCC |
| pJ204 backbone_fwd | USER PCR primer for donor plasmid (Backbone) | ACTTGCGUAGTGAGTCGAATAAGGGCGACACAAA |
| pJ204 backbone_rev | USER PCR primer for donor plasmid (Backbone) | ACACCGACUGAGTCGAATAAGGGCGACACCCCA |
| **5'/3' Junction PCR and Out-Out PCR** | | |
| COSMC genomic fwd [junction] | COSMC amplicon for 5' junction PCR | TGGTTTCTAGGCTAATGCTTTGA |
| COSMC genomic rev [junction] | COSMC amplicon for 3' junction PCR | CCTGCCCCCACAGAAAAGTA |
| Mgat1 genomic fwd [junction] – set1 | Mgat1 amplicon for 5' junction PCR | TAGACTGGGGAAGTGAGC |
| Mgat1 genomic rev [junction] – set1 | Mgat1 amplicon for 3' junction PCR | CAATAAAAGACGTCAGGAGGC |
| Mgat1 genomic fwd [junction] – set2 | Mgat1 amplicon for 5' junction PCR | TCTGCTGTCTCTACTGCCCT |
| Mgat1 genomic rev [junction] – set2 | Mgat1 amplicon for 3' junction PCR | CTATAGACACGGGCAAGGAA |
| LdhA genomic fwd [junction] | LdhA amplicon for 5' junction PCR | GGCTGCTGTTCAGAGGTCTT |
| LdhA genomic rev [junction] | LdhA amplicon for 3' junction PCR | TGAGGCTTACACACAGCACA |
| EF-1a rev [junction] | Amplicon for 5' junction PCR | ATCCTGGCCCGCATTTACAA |
| NeoR fwd [junction] | Amplicon for 3' junction PCR | CTGGACGAAGAGCATCAGGG |
| **Deep sequencing** | | |
| COSMC sgRNA2_F_Nex | COSMC amplicon for MiSeq analysis (sgRNA2 site) | TCGTCGGCAGCGTCAGATGTGTATAAGAGACAGTCCCACCTTGTTCAGGACACT |
| COSMC sgRNA2_R_Nex | COSMC amplicon for MiSeq analysis (sgRNA2 site) | GTCTCGTGGGCTCGGAGATGTGTATAAGAGACAGGGATCCATCGCAGCCTTTCTAT |
| Mgat1 sgRNA1_F_Nex | Mgat1 amplicon for MiSeq analysis (sgRNA1 site) | TCGTCGGCAGCGTCAGATGTGTATAAGAGACAGCCAAGGCCTTCTGGGATGAC |
| Mgat1 sgRNA1_R_Nex | Mgat1 amplicon for MiSeq analysis (sgRNA1 site) | GTCTCGTGGGCTCGGAGATGTGTATAAGAGACAGCGAACTGCTGGTTCAGCTTG |
| Mgat1 sgRNA2_F_Nex | Mgat1 amplicon for MiSeq analysis (sgRNA2 site) | TCGTCGGCAGCGTCAGATGTGTATAAGAGACAGGCGGGTACAGTACACTAGCAGA |
| Mgat1 sgRNA2_R_Nex | Mgat1 amplicon for MiSeq analysis (sgRNA2 site) | GTCTCGTGGGCTCGGAGATGTGTATAAGAGACAGAAGGCAGGTGCTGCTAATTCCA |
| Mgat1 sgRNA3_F_Nex | Mgat1 amplicon for MiSeq analysis (sgRNA3 site) | TCGTCGGCAGCGTCAGATGTGTATAAGAGACAGTGCAGCAAATCAGGGAGCAT |
| Mgat1 sgRNA3_R_Nex | Mgat1 amplicon for MiSeq analysis (sgRNA3 site) | GTCTCGTGGGCTCGGAGATGTGTATAAGAGACAGGAGGGCCGATAGTGCAACAA |
| Mgat1 sgRNA4_F_Nex | Mgat1 amplicon for MiSeq analysis (sgRNA4 site) | TCGTCGGCAGCGTCAGATGTGTATAAGAGACAGCCCCATCATTGTCAGCCGTC |
| Mgat1 sgRNA4_R_Nex | Mgat1 amplicon for MiSeq analysis (sgRNA4 site) | GTCTCGTGGGCTCGGAGATGTGTATAAGAGACAGGAACTTGTTGAAGATCTGGCCC |
| Mgat1 sgRNA5_F_Nex | Mgat1 amplicon for MiSeq analysis (sgRNA5 site) | TCGTCGGCAGCGTCAGATGTGTATAAGAGACAGTTCTGGACACGCCCAGC |
| Mgat1 sgRNA5_R_Nex | Mgat1 amplicon for MiSeq analysis (sgRNA5 site) | GTCTCGTGGGCTCGGAGATGTGTATAAGAGACAGGCCACGGTGGGCACTTT |
| LdhA sgRNA1_F_Nex | LdhA amplicon for MiSeq analysis (sgRNA1 site) | TCGTCGGCAGCGTCAGATGTGTATAAGAGACAGAAGTCCAAGATGGCAACACTCAA |
| LdhA sgRNA1_R_Nex | LdhA amplicon for MiSeq analysis (sgRNA1 site) | GTCTCGTGGGCTCGGAGATGTGTATAAGAGACAGCTCCCAATGCTTGGGCTTGTG |
| LdhA sgRNA2_F_Nex | LdhA amplicon for MiSeq analysis (sgRNA2 site) | TCGTCGGCAGCGTCAGATGTGTATAAGAGACAGGTGCTCTCCTGTGGAAACATTG |

| | | |
|---|---|---|
| LdhA sgRNA2_R_Nex | LdhA amplicon for MiSeq analysis (sgRNA2 site) | GTCTCGTGGGCTCGGAGATGTGTATAAGAGACAGAGTTTCCATGCTGCCAATCACG |
| LdhA sgRNA3_F_Nex | LdhA amplicon for MiSeq analysis (sgRNA3 site) | TCGTCGGCAGCGTCAGATGTGTATAAGAGACAGACAGCAAACTCCAAGCTGGT |
| LdhA sgRNA3_R_Nex | LdhA amplicon for MiSeq analysis (sgRNA3 site) | GTCTCGTGGGCTCGGAGATGTGTATAAGAGACAGCCAAGCCACGTAGGTCAAGA |
| LdhA sgRNA4_F_Nex | LdhA amplicon for MiSeq analysis (sgRNA4 site) | TCGTCGGCAGCGTCAGATGTGTATAAGAGACAGCTTGACCTACGTGGCTTGGA |
| LdhA sgRNA4_R_Nex | LdhA amplicon for MiSeq analysis (sgRNA4 site) | GTCTCGTGGGCTCGGAGATGTGTATAAGAGACAGTGATACTAAAGACTTACCACTGGAG |
| LdhA sgRNA5_F_Nex | LdhA amplicon for MiSeq analysis (sgRNA5 site) | TCGTCGGCAGCGTCAGATGTGTATAAGAGACAGTCTGCTCGATTCCGTTATCTGAT |
| LdhA sgRNA5_R_Nex | LdhA amplicon for MiSeq analysis (sgRNA5 site) | GTCTCGTGGGCTCGGAGATGTGTATAAGAGACAGGTTCTTAACAGAGCCATCTTTCT |
| COSMC OT1_F_Nex | COSMC amplicon for MiSeq analysis (sgRNA2 site: off-target1) | TCGTCGGCAGCGTCAGATGTGTATAAGAGACAGGTGTTCACAGCTGGGGCAT |
| COSMC OT1_R_Nex | COSMC amplicon for MiSeq analysis (sgRNA2 site: off-target1) | GTCTCGTGGGCTCGGAGATGTGTATAAGAGACAGGAAAGGGCAGGGGACTGAAA |
| COSMC OT2_F_Nex | COSMC amplicon for MiSeq analysis (sgRNA2 site: off-target2) | TCGTCGGCAGCGTCAGATGTGTATAAGAGACAGAGAGGGAGGGGATGCTAAGT |
| COSMC OT2_R_Nex | COSMC amplicon for MiSeq analysis (sgRNA2 site: off-target2) | GTCTCGTGGGCTCGGAGATGTGTATAAGAGACAGCCTTGGGTGTAAAGGCCCAT |
| COSMC OT3_F_Nex | COSMC amplicon for MiSeq analysis (sgRNA2 site: off-target3) | TCGTCGGCAGCGTCAGATGTGTATAAGAGACAGATTCAGGCCTGTTTGAGCCA |
| COSMC OT3_R_Nex | COSMC amplicon for MiSeq analysis (sgRNA2 site: off-target3) | GTCTCGTGGGCTCGGAGATGTGTATAAGAGACAGGGTAAAGGAGACCGCCATGT |
| COSMC OT4_F_Nex | COSMC amplicon for MiSeq analysis (sgRNA2 site: off-target4) | TCGTCGGCAGCGTCAGATGTGTATAAGAGACAGAGACATTGTCCACCCACAGC |
| COSMC OT4_R_Nex | COSMC amplicon for MiSeq analysis (sgRNA2 site: off-target4) | GTCTCGTGGGCTCGGAGATGTGTATAAGAGACAGGTGAGTGGAGGGTCTGGGAT |
| COSMC OT5_F_Nex | COSMC amplicon for MiSeq analysis (sgRNA2 site: off-target5) | TCGTCGGCAGCGTCAGATGTGTATAAGAGACAGGGAAAATACTACGTTATCCTTTGCT |
| COSMC OT5_R_Nex | COSMC amplicon for MiSeq analysis (sgRNA2 site: off-target5) | GTCTCGTGGGCTCGGAGATGTGTATAAGAGACAGCGTGGAGGAATAGCCTCTGT |
| Mgat1_sg1-OT1_F_Nex | Mgat1 amplicon for MiSeq analysis (sgRNA1 site: off-target1) | TCGTCGGCAGCGTCAGATGTGTATAAGAGACAGTGGTGTAGATTGTCCCACACCC |
| Mgat1_sg1-OT1_R_Nex | Mgat1 amplicon for MiSeq analysis (sgRNA1 site: off-target1) | GTCTCGTGGGCTCGGAGATGTGTATAAGAGACAGAAGACCTTTGGCTGGGATGTA |
| Mgat1_sg1-OT2_F_Nex | Mgat1 amplicon for MiSeq analysis (sgRNA1 site: off-target2) | TCGTCGGCAGCGTCAGATGTGTATAAGAGACAGGAAGCCATTGAGCTGCAAGTT |
| Mgat1_sg1-OT2_R_Nex | Mgat1 amplicon for MiSeq analysis (sgRNA1 site: off-target2) | GTCTCGTGGGCTCGGAGATGTGTATAAGAGACAGGCACTGAATGGGAAAAGGGG |
| Mgat1_sg5-OT1_F_Nex | Mgat1 amplicon for MiSeq analysis (sgRNA5 site: off-target1) | TCGTCGGCAGCGTCAGATGTGTATAAGAGACAGTGCGTGCAGCGTCCT |
| Mgat1_sg5-OT1_R_Nex | Mgat1 amplicon for MiSeq analysis (sgRNA5 site: off-target1) | GTCTCGTGGGCTCGGAGATGTGTATAAGAGACAGCGGAGGGTGGCCTGCT |
| Mgat1_sg5-OT2_F_Nex | Mgat1 amplicon for MiSeq analysis (sgRNA5 site: off-target2) | TCGTCGGCAGCGTCAGATGTGTATAAGAGACAGCTAAGGAAGAGCGTCAGATGGT |
| Mgat1_sg5-OT2_R_Nex | Mgat1 amplicon for MiSeq analysis (sgRNA5 site: off-target2) | GTCTCGTGGGCTCGGAGATGTGTATAAGAGACAGCGCCCTCGACTTTGACAGGA |
| Mgat1_sg5-OT3_F_Nex | Mgat1 amplicon for MiSeq analysis (sgRNA5 site: off-target3) | TCGTCGGCAGCGTCAGATGTGTATAAGAGACAGTGTACAACATGACTAATGGCAGC |
| Mgat1_sg5-OT3_R_Nex | Mgat1 amplicon for MiSeq analysis (sgRNA5 site: off-target3) | GTCTCGTGGGCTCGGAGATGTGTATAAGAGACAGCCTACCCCCACCCCGTG |
| Mgat1_sg5-OT4_F_Nex | Mgat1 amplicon for MiSeq analysis (sgRNA5 site: off-target4) | TCGTCGGCAGCGTCAGATGTGTATAAGAGACAGTTCATGGGCCTCATCCCCTA |

| | | |
|---|---|---|
| Mgat1_sg5-OT4_R_Nex | Mgat1 amplicon for MiSeq analysis (sgRNA5 site: off-target4) | GTCTCGTGGGCTCGGAGATGTGTATAAGAGAC AGGGGCACATTGGACTCAGACA |
| | **qRT-PCR** | |
| EF-1a - mCherry junction fwd | mCherry amplicon for qRT-PCR [Amplicon size: 100 bp] | CCTCAGACAGTGGTTCAAAGT |
| EF-1a - mCherry junction rev | mCherry amplicon for qRT-PCR [Amplicon size: 100 bp] | GATGGCCATGTTATCCTCCTC |
| COSMC fwd | COSMC amplicon for qRT-PCR [Amplicon size: 144 bp] | GCAGCCTTTCTATCTAGGACAC |
| COSMC rev | COSMC amplicon for qRT-PCR [Amplicon size: 144 bp] | CCACCTTGTTCAGGACACTT |
| LdhA fwd | LdhA amplicon for qRT-PCR [Amplicon size: 94 bp] | GAGTGAATGTAGCTGGTGTCTC |
| LdhA rev | LdhA amplicon for qRT-PCR [Amplicon size: 94 bp] | ACCTGCTTGTGAACCTCATT |
| Mgat1-sg1 fwd | Mgat1 amplicon for qRT-PCR (sgRNA1 site) [Amplicon size: 113 bp] | GGCCTGTATTCGTCCAGAAA |
| Mgat1-sg1 rev | Mgat1 amplicon for qRT-PCR (sgRNA1 site) [Amplicon size: 113 bp] | CGAACTGCTGGTTCAGCTTG |
| Mgat1-sg5 fwd | Mgat1 amplicon for qRT-PCR (sgRNA5 site) [Amplicon size: 128 bp] | CTCACCCGTGAGGTGTTC |
| Mgat1-sg5 rev | Mgat1 amplicon for qRT-PCR (sgRNA5 site) [Amplicon size: 128 bp] | GCCACGGTGGGCACTTT |
| Vinculin fwd | Vinculin amplicon for qRT-PCR [Amplicon size: 73 bp] | GCTGGTTGCTAAGAGGGAGG |
| Vinculin rev | Vinculin amplicon for qRT-PCR [Amplicon size: 73 bp] | ATCAGAGGCAGCTTTCACGG |
| Beta-actin fwd | Beta-actin amplicon for qRT-PCR [Amplicon size: 202 bp] | TTTCCAGCCTTCCTTCCTGGGTGA |
| Beta-actin rev | Beta-actin amplicon for qRT-PCR [Amplicon size: 202 bp] | TGGCATAGAGGTCTTTGCGGATGT |

**Supplementary Table 3.** Plasmids used in this study as PCR templates

| Plasmid name | Elements | Source |
|---|---|---|
| pBudCE4.1 | EF-1a | Life Technologies |
| pmCherry-N1 | mCherry | Clontech |
| pcDNA3.1(+) | pCMV, BGH pA, pSV40- NeoR – SV40 pA | Life Technologies |
| pZsGreen1-DR | ZsGreen1-DR | Clontech |
| pU0020 | AmpR, pUC19 replication origin | Lund et al., 2014 |
